# Enrichment of an intraspecific genetic map of upland cotton by developing markers using parental RAD sequencing

**DOI:** 10.1093/dnares/dsu047

**Published:** 2015-02-05

**Authors:** Hantao Wang, Xin Jin, Beibei Zhang, Chao Shen, Zhongxu Lin

**Affiliations:** National Key Laboratory of Crop Genetic Improvement, Huazhong Agricultural University, Wuhan 430070, Hubei, China

**Keywords:** *Gossypium hirsutum*, RAD sequencing, genetic map, QTL mapping, comparative genomics

## Abstract

RAD sequencing was performed using DH962 and Jimian5 as upland cotton mapping parents. Sequencing data for DH962 and Jimian5 were assembled into the genome sequences of ≈55.27 and ≈57.06 Mb, respectively. Analysing genome sequences of the two parents, 1,323 SSR, 3,838 insertion/deletion (InDel), and 9,366 single-nucleotide polymorphism (SNP) primer pairs were developed. All of the SSRs, 121 InDels, 441 SNPs, and other 6,747 primer pairs were screened in the two parents, and a total of 535 new polymorphic loci were identified. A genetic map including 1,013 loci was constructed using these results and 506 loci previously published for this population. Twenty-seven new QTLs for yield and fibre quality were identified, indicating that the efficiency of QTL detection was greatly improved by the increase in map density. Comparative genomics showed there to be considerable homology and collinearity between the A_T_ and A_2_ genomes and between the D_T_ and D_5_ genomes, although there were a few exchanges and introgressions among the chromosomes of the A_2_ genome. Here, the development of markers using parental RAD sequencing was effective, and a high-density intraspecific genetic map was constructed. This map can be used for molecular marker-assisted selection in cotton.

## 1. Introduction

Among the four cultivated species (two diploids, *Gossypium herbaceum* and *Gossypium arboreum*, and two allotetraploids, *Gossypium hirsutum* and *Gossypium barbadense*) of cotton, upland cotton (*G. hirsutum*, 2*n* = 4*x* = 52, genome size ≈2.5 Gb) is widely cultivated in the world and accounts for ∼95% of worldwide cotton production.^1^ However, the narrow genetic background of upland cotton has resulted in inbreeding depression and reduced genetic variability, and the improvement of cotton yield was slow using conventional cultivar breeding programmes. The development of molecular marker technology provides an opportunity for the construction of a genetic linkage map. A high-density genetic map creates a better platform for researching the structure of an organism's genome, for dissecting traits of interest, for fine mapping of QTLs, and for map-based cloning.^2^

There are 19,074 publicly available SSRs (genomic SSRs and EST-SSRs), 3,541 RFLPs, 2,146 AFLPs, and 1,018 single-nucleotide polymorphisms (SNPs) on the CottonGen database (http://www.cottongen.org/), and most of them have been used to construct interspecific and intraspecific genetic maps. However, due to the narrow genetic diversity of upland cotton, the polymorphism of molecular markers between intraspecific hybrids of upland cotton is low. To date, due to the number of markers and the genome coverage, upland cotton genetic maps are inferior to interspecific maps.^3–6^ The development of more polymorphic markers is thus needed.

SNPs are single-base variations, including transitions, transversions, and insertions/deletions (InDels). SNPs are the most abundant type of molecular genetic markers in the genome, can be found in coding and non-coding regions,^7^ and are used for genetic map construction, genetic diversity analysis, QTL mapping, and marker-assisted selection breeding.^8^ The rapid development of next-generation sequencing (NGS) technology has facilitated genome-wide SNP discovery, and studies of NGS-derived SNPs have been reported in diploid and complex polyploid plants such as common bean,^8^ rice,^9^ and sorghum.^10^ The application of NGS-derived SNPs in cotton was rare. Byers *et al.*^11^ detected a larger number of SNPs generated from the GR-RSC libraries by using the Roche 454 pyrosequencing platform, in which 11,834 SNPs were found in 6,469 contigs between two *G. hirsutum*, Acala Maxxa and TX2094. As one of several methods that are based on NGS platforms to develop markers,^12^ restriction site-associated DNA sequencing (RAD-Seq) obtains sequences of restriction enzyme digestion tags using Illumina sequencing.^13^ This technology can greatly reduce the complexity of genomes, can identify abundant genetic markers quickly in an entire genome of some species with and without a reference genome, and also combines the advantages of low cost and high throughput.^14^ Bus *et al.*^15^ detected >20,000 SNPs and 125 InDels from >113,000 RAD clusters of *Brassica napus*, and about one-third of the RAD clusters were mapped on the reference sequence of *Brassica rapa*. The RAD-seq has been used to generate large numbers of SNPs for many species recently.^15–17^ This method can be applied in upland cotton to explore and develop more genetic markers to promote genome research in this species.

In this study, two RAD libraries using two mapping parents of upland cotton were constructed. The objectives were to (i) develop molecular markers based on parental RAD sequencing, (ii) enrich a high-density *G. hirsutum* genetic map using these molecular markers, (iii) detect new QTLs associated with yield components and fibre quality traits, and (iv) assess the homology of *G. hirsutum* to the A_2_ and D_5_ genomes.

## 2. Materials and methods

### 2.1. Plant materials and field trait data collection

*Gossypium hirsutum* acc. DH962 and *G. hirsutum* cv. Jimian5 served as the parents of an F_2_ mapping population.^3^ This mapping population was used to screen for polymorphisms and to construct a genetic map.

The traits of F_2_ individuals were represented by the average values of F_2:3_ lines.^18^ The traits included the number of bolls per plant (BN), seed cotton weight per boll (SCW), lint weight per boll (LW), lint percentage (LP), lint index (LI), seed index (SI), fibre length (FL, mm), fibre strength (FS, cN/tex), fibre length uniformity ratio (FU), fibre elongation (FE), and fibre micronaire (MV).

### 2.2. RAD library preparation and sequencing

Fresh young leaf tissue from the two parents was used to extract the genomic DNA using a Plant Genomic DNA Kit (TIANGEN Biotech, Beijing, China) in accordance with the manufacturer's instructions. Each of the two DNA samples was processed into RAD libraries in a manner similar to that reported by Baird *et al.*^14^ Briefly, genomic DNA was digested for 60 min at 37°C in a 50 μl reaction with 20 units (U) of *Eco*RI (New England Biolabs, NEB), and then the samples were heat inactivated at 65°C for 20 min. Then 2.5 μl of 100 nM P1 adapter, a modified Illumina adapter (Illumina, Inc.), was added to each sample along with 1 μl of 10 mM ATP (Promega), 1 μl of 10× NEB Buffer 4, 1 μl of 1,000 U of T4 DNA ligase (Enzymatics, Inc.), and 5 μl H_2_O, and the reaction was incubated at room temperature for 20 min. Samples were again heat inactivated for 20 min at 65°C, pooled, and randomly sheared with a Bioruptor (Diagenode) to an average size of 500 bp. Samples were then separated by electrophoresis through a 1.5% agarose, 0.5× TBE gel, and DNA fragments from 300 to 700 bp were isolated using a MinElute Gel Extraction Kit (Qiagen). End blunting enzymes (Enzymatics, Inc.) were used to polish the dsDNA ends. The samples were repurified using a MinElute column (Qiagen). Then 15 U of Exo-Klenow (Enzymatics, Inc.) was added, and the sample was incubated at 37°C to generate 3′ adenine overhangs. The samples were purified, and 1 μl of 10 μM P2 adapter, a divergent modified Solexa adapter (Illumina, Inc.), was ligated to these DNA fragments at 18°C. The samples were again purified as above and eluted in 50 μl. The eluate was quantified using a Qubit fluorimeter, and 20 ng of this product was used in PCR amplification with 20 μl Phusion MasterMix (NEB), 5 μl of 10 μM modified Solexa Amplification primer mix (Illumina, Inc.), and up to 100 μl H_2_O. Phusion PCR settings were copied from the product guidelines, and the samples were processed for a total of 18 cycles. Samples were gel purified, and excised DNA ranged from 300 to 700 bp in size. It was diluted to 1 nM.

The two RAD libraries were run on an Illumina Genome Analyzer II at Beijing Genomics Institute (BGI) in Shenzhen. Illumina/Solexa protocols were followed for a 2 × 50 base paired-end (PE) sequencing run.

### 2.3. Sequence analysis and *de novo* assembly

Raw reads of the two materials were filtered to remove reads with the following conditions: >2% *N* calls, polyA structures, adapter contamination, base (quality scores ≤ 5) number accounts for 50% of the reads in PE libraries. A FASTX Toolkit Updated (http://hannonlab.cshl.edu/fastx_toolkit/) was used to filter the PE reads further, and then the sequences were assembled using a Velvet sequence assembler (version 1.0.18).^19^ This system was with a hash length of 31 bp and a minimum contig size of 200 bp, with other parameters set to default values.

CAP3^20^ was used to identify sequences in common between the mapping parents, with the overlap length cut-off set at 80 bp and overlap percent cut-off set at 95. The resulting non-redundant data set (Gh-D-J; *Gossypium hirsutum*-DH962-Jimian5) included singletons from ‘DH962’ and ‘Jimian5’ and common contigs derived from both parents' assembled contigs.

### 2.4. Sequence annotation

A BLASTX search was performed against the TAIR10 protein databases (http://www.arabidopsis.org/) with an *E*-value cut-off of 1e−15 for the sequences of the Gh-D-J data set. The annotated sequences were assigned functions based on Arabidopsis GO SLIM (ftp://ftp.arabidopsis.org/home/tair/Ontologies/Gene_Ontology/) and then mapped to higher level categories (plant GO slim) using GOSlim Viewer^21^ according to the three principal GO categories: molecular function, cellular component, and biological process.^22^

### 2.5. Mining of SSRs, InDels, and SNPs and primer design

**Figure 1.**
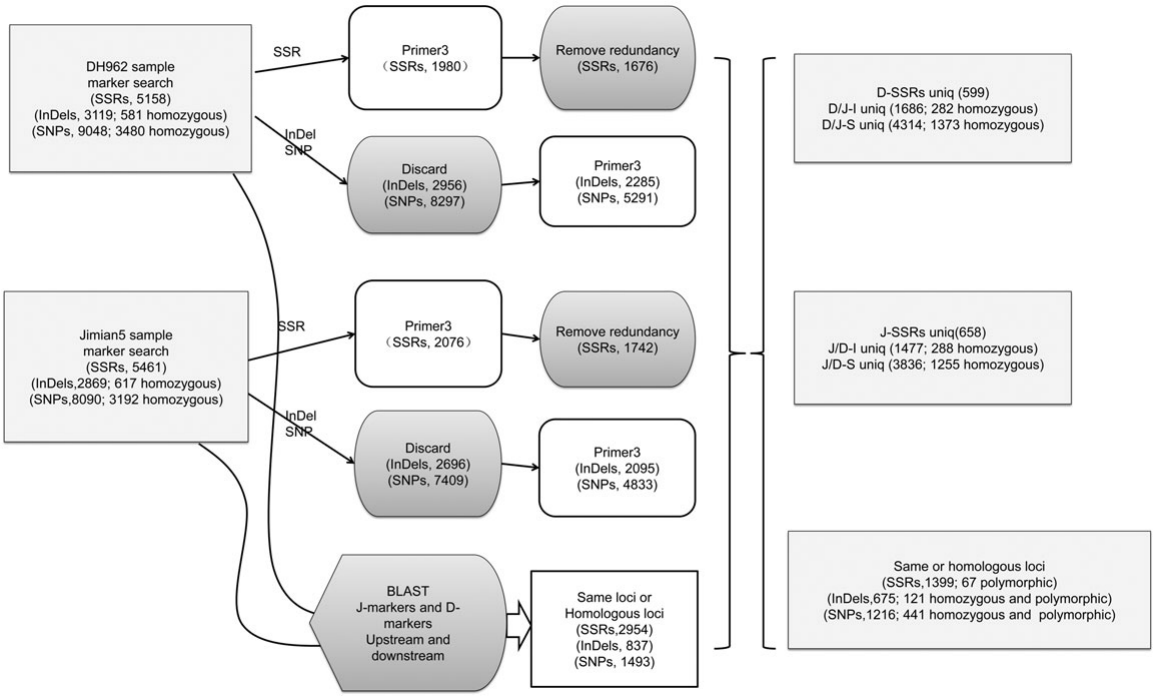
Overall workflow of marker discovery and analysis.

An overall workflow of marker discovery and analysis performed in this study is shown in Fig. 1.

#### 2.5.1. SSR

The contigs of two parents were screened for SSR motifs using the microsatellite (MISA) searching tool (http://pgrc.ipk-gatersleben.de/misa/) implemented in PERL. The parameter settings were as follows: the minimum repeat unit was defined as seven repeats for dinucleotide motifs, five repeats for tri-motifs, four repeats for tetra-motifs, and three repeats for penta-, hexa-, hepta-, and octa-motifs. The maximum number of interrupting bases in a compound microsatellite was set to 500.

#### 2.5.2. InDel

BWA (http://sourceforge.net/projects/bio-bwa/files/) was used to map the RAD sequencing reads of DH962 sample to the assembled contigs of Jimian5 and map the RAD sequencing reads of Jimian5 sample to the assembled contigs of DH962. The mapping results were transformed into bam format with SAMtool v0.1.19.^23^ Then the bam files were sorted, and duplicates were removed using SAMtool v0.1.19. Finally, GATK software was used to call InDels.^24^

#### 2.5.3. SNP

SOAPaligner v2.21 (http://soap.genomics.org.cn/soapaligner.html) software was used to map the RAD sequencing reads of DH962 to the assembled contigs of Jimian5 and map the RAD sequencing reads of Jimian5 to the assembled contigs of DH962, run with a maximum mismatch of 2 bp. The mapping results, shown as RAD SE reads, were used to identify SNPs using SOAPsnp.^25^

For all SNPs and InDels mined, sites were considered as homozygous when the minor allele frequency was below 0.10, heterozygous when the minor allele frequency was above 0.25, and unknown when the minor allele frequency was between 0.10 and 0.25.

The contigs containing markers were used to design primers, employing the Primer3 program (http://bioinfo.ut.ee/primer3/). The primer length was set between 18 and 24 nucleotides with an optimum size of 20 bp; the optimum annealing temperature was 57°C; the GC content was set between 40 and 60% with an optimum GC content of 50%; and the predicted PCR products ranged from 100 to 500 bp. If the distance between two or more markers was <500 bp in a region, primers were for both ends of that region. However, the predicted PCR product was shorter than 800 bp in this study.

### 2.6. Remove the redundancy of SSRs

A total of 13 Mb of sequence data (genomic SSRs and EST-SSRs) were collected from all the SSR markers of the CottonGen database and used to assess the novelty of these SSR sequences. Among the collected markers, some were assembled by several ESTs using the Phrap program (http://www.phrap.org/index.html). Because most of the collected markers were from ESTs, and RAD sequences were genomic, it was necessary to remove the redundant sequences accurately. This was accomplished as follows: (i) the sequences between the SSR primers were extracted from RAD sequences; (ii) the SSRs in extracted sequences were shielded using RepeatMask (http://www.repeatmasker.org/) program; (iii) the remaining of sequences, which lacked SSRs, were BLAST against the collected markers; if the matched bases numbered ≥50, the SSR primers were considered to be redundant.

### 2.7. Marker redundancy check between DH962 and Jimian5

**Figure 2.**
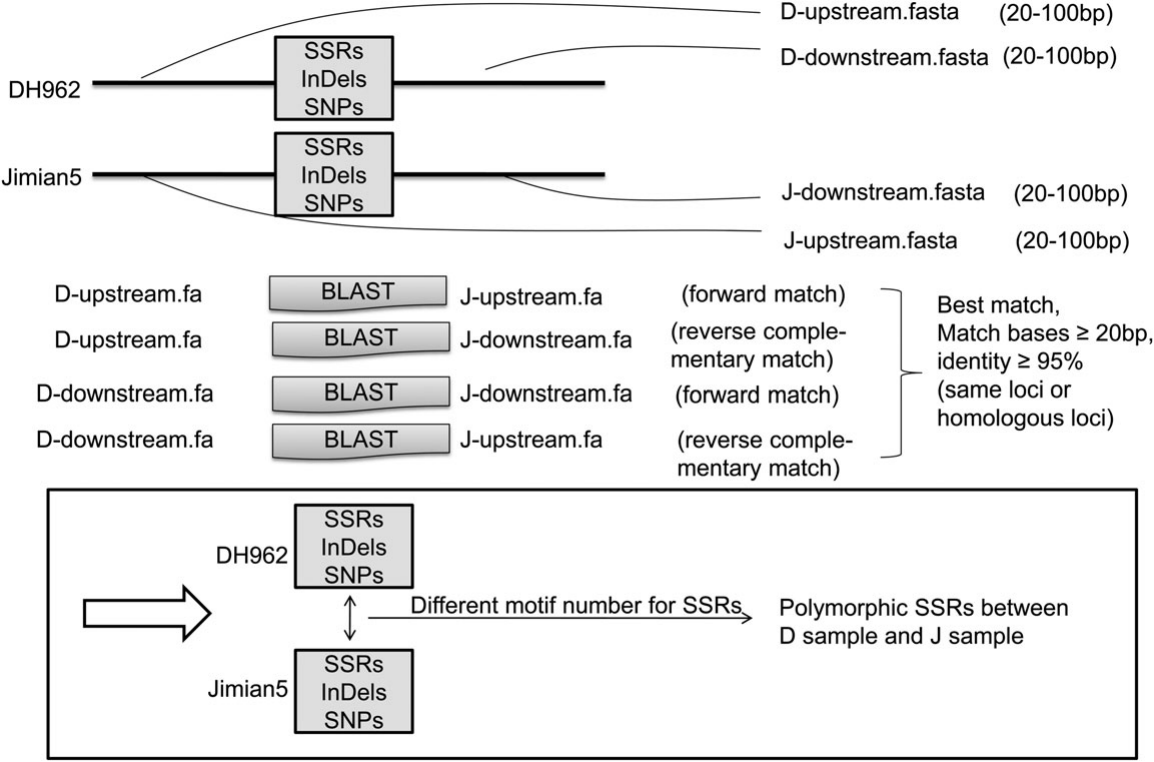
Comparative analysis of the flanking sequences of markers of DH962 and Jimian5.

Among markers between DH962 and Jimian5, some were located on the same location or homologous location. We used the immediate flanking sequences (≥20 bp on both sides) of Jimian5 markers to BLAST the immediate flanking sequences (≥20 bp on both sides) of DH962 markers with an *E*-value cut-off of 1e−5 (Fig. 2).

Finally, the remaining markers with primers were given the prefix HAU (indicating Huazhong Agricultural University) and synthesized by Beijing Tianyi Huiyuan Life Science and Technology, Inc. (Wuhan, China).

### 2.8. Homology of the upland cotton to the A_2_ and D_5_ genomes

For further analysis of the homology of the two sub-genomes of upland cotton to the A_2_ and D_5_ genomes, a BLASTN search was performed against the diploid A_2_ genome of *Gossypium arboretum* and D_5_ genome of *Gossypium raimondii* (http://www.phytozome.net/cotton.php) with an *E*-value cut-off of 1e−10 for the sequences of primers on the upland cotton genetic map in this study.

### 2.9. Marker genotyping

In addition to the primers developed in this study, a total of 1,869 markers were also chosen according to the criteria published by Wang^26^ and an interspecific BC_1_ genetic map from this lab.^27^ A total of 4,878 SSRs (3,479 HAU, 699 NAU, 700 Gh) were obtained from previous studies.^28–34^ PCR amplifications of primers and silver staining were performed as described by Lin *et al.*^3^ PCR products of all the primer pairs were separated on 6% denaturing polyacrylamide gel^3^ or 8% native polyacrylamide gels using single-strand conformation polymorphism (SSCP) technology.^26^ The SSCP technology workflow was as follows: PCR products were run on 8% native polyacrylamide gels (1:29, bis to acrylamide). The gels were exposed to electrophoresis at a constant 15 W at 25°C for 3.5–4 h. After electrophoresis, the DNA fragments were visualized by silver staining. The silver staining protocol was the same as the SSR protocol.

### 2.10. Linkage map construction and QTL analysis

A *χ*^2^ test was performed to determine whether the genotypic frequencies at each locus deviated from the expected 1 : 2 : 1 or 3 : 1 segregation ratio in the F_2_ population. The polymorphic loci were integrated into the F_2_ linkage map using JoinMap V3.0.^35^ The logarithm of odds (LOD) threshold was 4.0, and the maximum recombination fraction was 0.4. Genetic map distances in centiMorgans (cM) were calculated using the Kosambi mapping function. The resulting linkage map was drawn using MapChart V2.2 software.^36^ Linkage groups were assigned to chromosomes on the basis of a BC_1_ linkage map,^27^ an F_2_ linkage map,^3^ and marker mapping information on the CottonGen database.

QTLs were identified using Windows QTL Cartographer 2.5 (http://statgen.ncsu.edu/qtlcart/WQTLCart.htm) by composite interval mapping (CIM). The statistical significance of the LOD threshold value was determined by running a permutation procedure 1,000 times for all traits. QTL nomenclature was adapted using the method proposed by McCouch *et al.*^37^

## 3. Results

### 3.1. RAD sequencing and *de novo* contig assembly

A total of 62.46 million and 61.27 million raw reads were produced from the DH962 and Jimian5 RAD libraries, respectively. After quality filtering, 5.15 and 5.18 Gb of clean reads were obtained from DH962 and Jimian5 RAD libraries, the GC contents were 34.00 and 34.17%, and the Q scores >20 were 97.84 and 97.94%, respectively.

Initial *de nov*o assembly produced ≈55.27 Mb of DH962 genome sequence distributed over 178,157 individual contigs. Contig lengths ranged from 200 to 3,195 bp with an average length of 310 bp, and the lengths of most contigs were between 200 and 800 bp. The clean reads of Jimian5 were assembled into a ≈57.06 Mb genome sequence distributed over 181,422 individual contigs. Contig lengths ranged from 200 to 2,355 bp with an average length of 316 bp, and the lengths of most contigs were between 200 and 800 bp.

**Figure 3.**
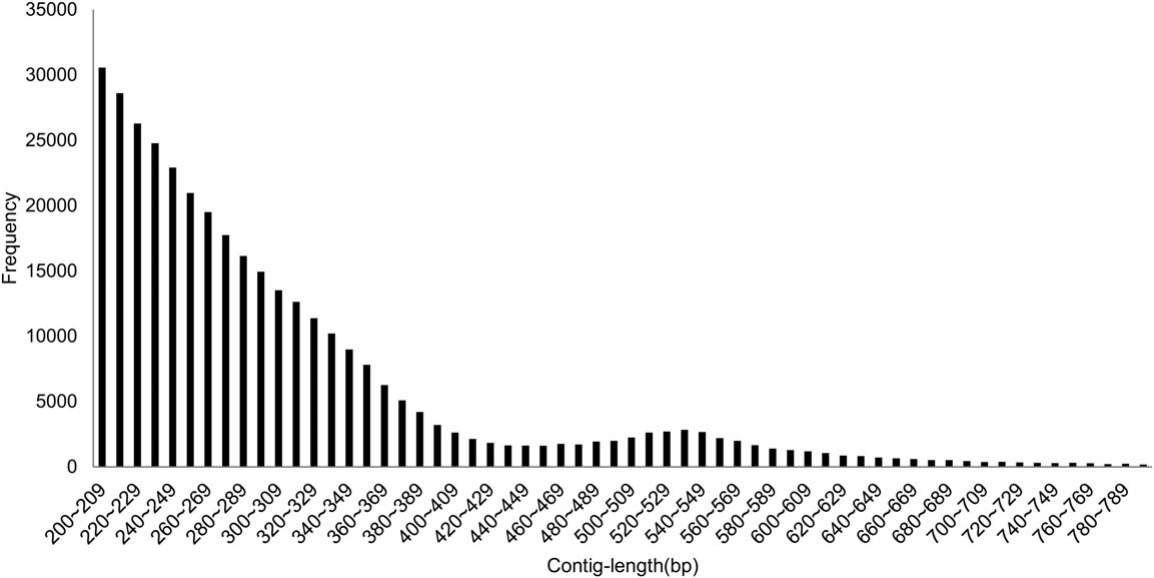
Sequence length distribution of the Gh-D-J data set.

**Figure 4.**
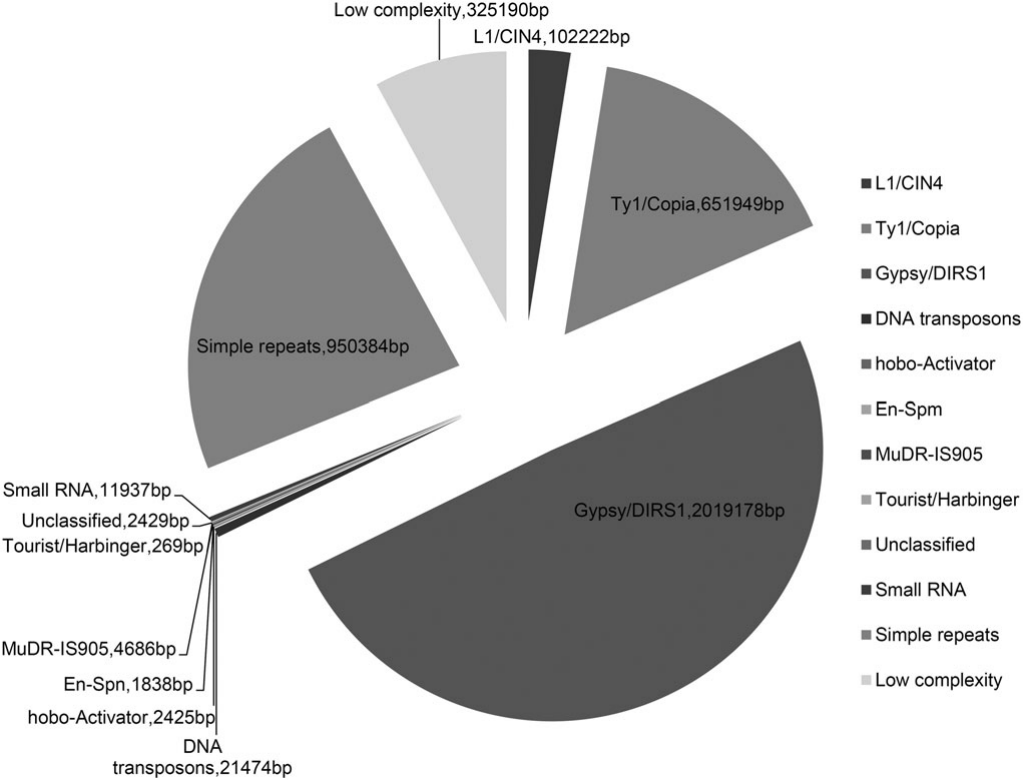
The representation of known repetitive elements in *Gossypium hirsutum* RAD sequences.

The Gh-D-J data set consisted of 251,816 sequences totalling ≈85.76 Mb (mean length 340 bp, Fig. 3), of which 105,264 (38.14 Mb) sequences were shared (Table 1). The repetitive elements in the sequences, Gh-D-J, were evaluated using the RepeatMasker (http://www.repeatmasker.org/). The relative number of repetitive elements on the contigs was 4.76%, which is similar to results from PE RAD-seq studies in other plant genomes.^17,38^ The major classes of repetitive DNA elements belonged to Gypsy/DIRS1 and Ty1/Copia long-terminal repeat (LTR) retroelement families and simple repeats (Fig. 4). The GC dinucleotide content for Gh-D-J was ∼34.72%, which is similar to that of both *Arabidopsis thaliana*^39^and *Theobroma cacao*.^39,40^

**Table 1.** Summary statistics of the RAD tag sequencing via illumina (DH962, Jimian5)

Feature	DH962	Jimian5
Illumina reads (million) after sequence editing	62.46	61.27
Sequences bases (Gb) after sequence editing	5.15	5.18
Number of contigs	178,157 (55.27 Mb)	181,422 (57.06 Mb)
Average contig length (bp)	310	314
Common contigs	105,264 (38.14 Mb)	
Contig number of Gh-D-J data set	251,816 (85.76 Mb)	
Number of SSR primer pairs	1,323	
Number of InDel primer pairs	3,838	
Number of SNP primer pairs	9,366	
Number of sequences with markers	14,433

### 3.2. GO categorization

**Figure 5.**
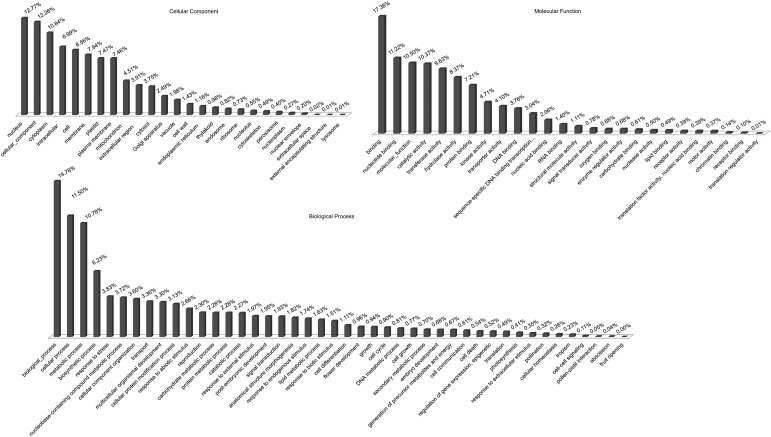
Gene ontology classification of the Gh-D-J data set. The three GO categories are cellular component, molecular function, and biological process.

In all, 15,535 (6.17%) sequences of the Gh-D-J data set were significantly matched to 7,719 *A. thaliana* genes (Supplementary Table S1). The annotated Gh-D-J sequences were further functionally classified using a plant-specific GO slim (Fig. 5). Of the Gh-D-J sequences, 41,222 GO terms were categorized under biological process, 19,406 under cellular component, and 13,899 under molecular function. Fibre development is a very important process in cotton. Then 58 contigs were identified as being related to the MYB family, 10 contigs were found to be related to expansin biosynthesis, and 27 contigs were found to be related to the ethylene response. A total of 60 contigs were related to cell wall and cellulose biogenesis, 17 of which were related to cellulose synthase (*CESA*) genes (16 were required in expanding primary walls, and 1 was required in the thickening of secondary walls), 11 to the cellulose synthase protein family, and 32 to cellulose synthase-like (*CSL*) genes.

The research and discovery of pest- and disease-resistant genes is also important. A total of 46 contigs were found to be related to nucleotide-binding site (NBS) domains using GO analysis, 15 contigs were found to be related to the coiled-coil (CC)-NBS-leucine-rich repeat (LRR) family, 12 contigs were related to the toll/interleukin-1 receptor (TIR)-NBS family, and 19 contigs were related to the TIR-NBS-LRR family.

### 3.3. Characterization and identification of SSRs in the two RAD libraries

A total of 5,158 SSRs (containing 244 compound SSRs) and 5,461 SSRs (containing 246 compound SSRs) were identified within contig sequences of DH962 and Jimian5, respectively, with an average frequency of 1/10.58 kb (Table 2). In the two RAD libraries, the most abundant type of repeat motifs was pentanucleotide repeat (Table 2). The frequencies of motif types were similar (Table 2), with AT/TA as the most frequent repeat, followed by AAAAT/ATTTT, AAT/ATT, AAAT/ATTT, AAG/CTT, and AAAAG/CTTTT in descending order (Table 2).

**Table 2.** Summary of SSRs identified from two libraries

Research items	DH962	Jimian5
Total number of identified SSRs	5,158	5,461
Number of SSR-containing sequences	4,911	5,214
Number of SSRs present in compound formation	244	246
Frequency of SSRs	1/10.72 kb	1/10.44 kb
Frequency of motif size	Dinucleotide (18.96%)	Dinucleotide (18.57%)
	Trinucleotide (17.82%)	Trinucleotide (18.49%)
	Tetranucleotide (12.39%)	Tetranucleotide (12.54%)
	Pentanucleotide (33.29%)	Pentanucleotide (32.72%)
	Hexanucleotide (13.90%)	Hexanucleotide (13.94%)
	Heptanucleotide (3.16%)	Heptanucleotide (3.19%)
	Octanucleotide (0.48%)	Octanucleotide (0.55%)
Frequency of major motif type	AT/AT (12.62%)	AT/AT (12.43%)
	AAAAT/ATTTT (12.04%)	AAAAT/ATTTT (11.79%)
	AAT/ATT (7.06%)	AAT/ATT (7.34%)
	AAAT/ATTT (6.07%)	AAAT/ATTT (6.08%)
	AAG/CTT (5.16%)	AAAAG/CTTTT (5.44%)
	AAAAG/CTTTT (4.94%)	AAG/CTT (5.13%)
	AG/CT (3.78%)	AG/CT (3.77%)
	AAATT/AATTT (3.70%)	AAATT/AATTT (3.70%)
	AAAAAT/ATTTTT (2.77%)	AAAAAT/ATTTTT (3.19%)
	AC/GT (2.56%)	ATC/ATG (2.44%)
	ATC/ATG (2.27%)	AC/GT (2.36%)
	AATAT/ATATT (1.78%)	AAAAAG/CTTTTT (1.50%)
	AATT/AATT (1.43%)	AATAT/ATATT (1.46%)
	ACAT/ATGT (1.40%)	AATT/AATT (1.39%)
	AAAAAG/CTTTTT (1.40%)	ACAT/ATGT (1.37%)
	AAAAC/GTTTT (1.28%)	AAAG/CTTT (1.28%)
	AAAG/CTTT (1.22%)	AAAAC/GTTTT (1.26%)
	AAC/GTT (1.07%)	AAC/GTT (0.97%)

After redundancies and duplications were removed, 1,399 non-redundant SSRs remained in both DH962 and Jimian5. These were located on the same location or homologous location. Among the 1,399 SSRs (Supplementary Table S2a), there were 67 SSRs (Supplementary Table S2b) that were different in the number of motifs between DH962 and Jimian5, which were *in silico* polymorphic. A comparison of the SSR sequences of DH962 and Jimian5 showed 598 (Supplementary Table S2c) non-redundant SSRs in DH962 only and 658 (Supplementary Table S2d) non-redundant SSR in Jimian5 only. Finally, 1,323 SSR primer pairs (67 + 598 + 658) were used to screen for polymorphisms (Fig. 1).

### 3.4. Characterization and identification of InDels and SNPs in the two RAD libraries

**Figure 6.**
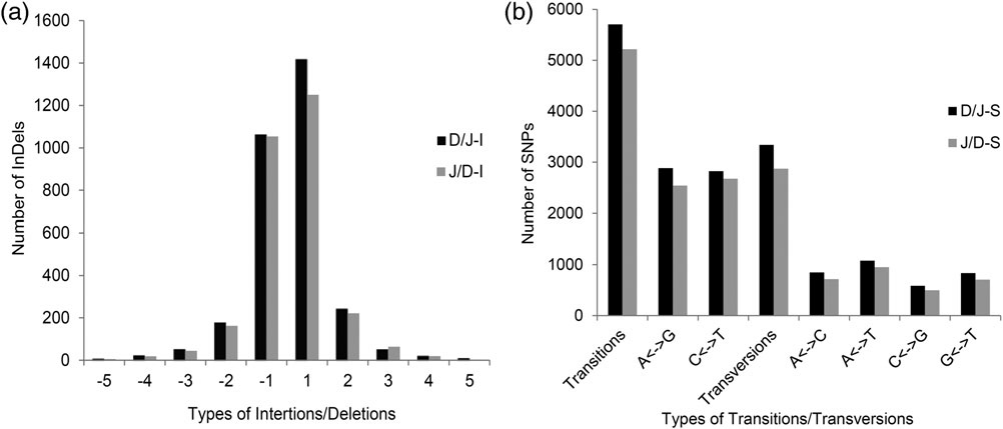
Characteristics and distribution of InDels and SNPs in two RAD libraries. (a) Distribution of insertions (+) and deletions (−). (b) Transitions and transversions occurring within the mined SNPs.

InDel calling showed there to be 3,558 InDels after mapping DH962 reads to Jimian5 assembled contigs (D/J-I) and 3,240 InDels after mapping Jimian5 reads to DH962 assembled contigs (J/D-I). The called results that conformed to any one of the conditions {MQ0 ≥ 4 [[MQ0/(1.0 * DP)] > 0.1]; MQ < 30.0; QUAL < 50; DP < 5} were removed. After filtering, the mapping of D/J-I included 3,119 InDels, and the mapping of J/D-I included 2,869 InDels. The frequency of InDels was 1/18.46 kb. Among the 3,119 InDels of D/J-I, 1,355 were insertions and 1,764 were deletions, with the average length of the InDels being 1.4 bp (Fig. 6a). Among the 2,869 InDels of J/D-I, 1,301 were insertions, and 1,568 were deletions. The average length of the InDels was 1.3 bp (Fig. 6a). After BLAST of the flanking sequences of InDels, 1,686 InDels were found to appear only in D/J-I (Supplementary Table S3a), and 1,477 InDels appeared only in J/D-I (Supplementary Table S3b), with 675 InDels appearing in both (Supplementary Table S3c). Among the 675 sites, there were 121 sites between DH962 and Jimian5 that were homozygous and polymorphic (Supplementary Table S3d). A total of 3,838 InDel primer pairs were obtained (Fig. 1).

SNP calling identified 26,757 SNPs in D/J-S and 25,682 SNPs in J/D-S. When screened with a coverage depth ≥ 8, 9,048 SNPs remained in D/J-S, and 8,090 SNPs remained in J/D-S. The frequency of SNPs was 1/6.55 Kb, and the transition/transversion ratio of all the SNPs was 1.76. Of the 9,048 SNPs in D/J-S, the observed SNP transition/transversion ratio was 1.71 (Fig. 6b), and most SNPs were identified between 50 and 350 bp of the start of each contig. Of the 8,090 SNPs in J/D-S, the observed SNP transition/transversion ratio was 1.82, and most SNPs were identified between 50 and 300 bp from the start of each contig.

After BLAST the flanking sequences of SNPs, 4,314 SNPs were found to appear only in D/J-S (Supplementary Table S4a), and 3,836 SNPs appeared only in J/D-S (Supplementary Table S4b), with 1,216 SNPs being found in both (Supplementary Table S4c). Of the 1,216 sites, there were 441 sites that were homozygous and polymorphic between DH962 and Jimian5 (Supplementary Table S4d). Finally, a total of 9,366 SNP primer pairs were obtained (Fig. 1).

### 3.5. *In silico* mapping of the marker contigs

**Figure 7.**
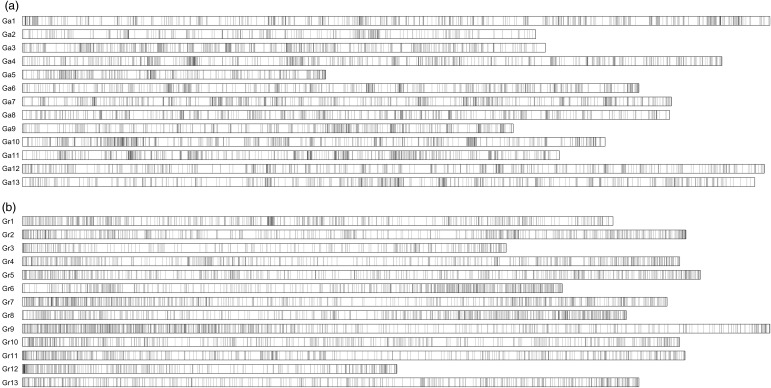
The distribution of the 14,103 contigs on the A_2_ and D_5_ genomes. (a) The distribution of contigs on the A_2_ genome; (b) the distribution of contigs on the D_5_ genome.

A total of 14,433 contigs containing markers were used to analyse the distribution of the markers on the A_2_ and D_5_ genomes. We used all of the 14,433 contigs to BLAST with the sequences of the A_2_ and D_5_ genomes, and 14,103 (97.71%) contigs were matched (Fig. 7). Of the 14,103 contigs, 6,995 were matched on the 13 chromosomes of the A_2_ genome, and 7,108 were matched on the 13 chromosomes of the D_5_ genome. The hits were found in an essentially uniform distribution on every chromosome, except there were slightly fewer on the Ga2 and Ga5 of the A_2_ genome and on Gr3 and Gr12 of the D_5_ genome.

### 3.6. Validation of the SSR, InDel, and SNP markers

All of the 1,323 SSRs were screened for polymorphisms between DH962 and Jimian5. After being genotyped on 6% denaturing polyacrylamide gels, 1,304 (98.56%) were amplified successfully, and 66 SSRs showed polymorphism. The remaining 1,257 SSRs were subjected to SSCP analysis on 8% native polyacrylamide gels, and 17 SSRs showed polymorphisms. A total of 83 SSRs showed polymorphisms (6.27%). Among the 67 SSRs that were polymorphic between DH962 and Jimian5 by bioinformatic analysis, 12 (17.91%) showed polymorphisms.

Of the 3,838 InDels and 9,366 SNPs, 121 InDels and 441 SNPs, which were *in silico* polymorphic between two parents, were selected to detect polymorphisms between DH962 and Jimian5 by SSCP analysis. Thirty-one InDels (25.62%) were polymorphic and revealed 33 polymorphic loci, and 49 SNPs (11.11%) were polymorphic.

The polymorphisms of the 1,323 SSRs, 121 InDels, and 441 SNPs between *G. hirsutum* cv. Emian22 and *G. barbadense* acc. 3–79, the mapping parents of our interspecific population,^41^ were also screened. A total of 610 SSRs (46.11%) (437 were genotyped on 6% denaturing polyacrylamide gels, and 173 were analysed by SSCP), 46 InDels (38.02%), and 127 SNPs (28.80%) were found to be polymorphic.

### 3.7. Genetic linkage map construction

A total of 3,479 HAU, 699 NAU, and 700 Gh SSR primer pairs obtained from previous studies^28–34^ were screened for polymorphism in DH962 and Jimian5, and 173 (3.55%) showed polymorphisms, revealing 178 polymorphic loci. Additionally, 1,869 markers from the current interspecific BC_1_ genetic maps^27^ and Wang *et al.*^26^ were screened for polymorphism in DH962 and Jimian5, and 187 showed polymorphisms, revealing 192 polymorphic loci. Adding the 165 polymorphic loci obtained from this study and the previously published 506 loci,^3^ a total of 1,041 loci were used for linkage analysis. Finally, 1,013 loci were mapped on 50 linkage groups with 41 linkage groups assigned to 23 chromosomes (Supplementary Fig. S1). The total length of the linkage map was 3,004.71 cM, with a mean distance of 2.97 cM between adjacent markers.

Among the 535 loci obtained in this study, 99 loci deviated from an expected 1 : 2 : 1 or 3 : 1 segregation ratio (*P* ≤ 0.05), and 8 loci were found to not be mapped. Seven segregation distortion regions were found on four chromosomes, with many segregation distortion loci found on LG1/Chr9 or 23, LG5/Chr7, and LG6/Chr25.

### 3.8. QTLs for yield components and fibre quality traits

For yield components, 21 QTLs were detected and mapped on seven chromosomes (Supplementary Fig. S1), and explained from 9.80 to 16.62% of the phenotypic variation (PV), with LOD scores ranging from 4.22 to 7.83 (Supplementary Table S5). There were 2 QTLs for BN, 2 for SCW, 6 for LW, 5 for SI, 2 for LP, 4 for LI, and 18 were newly found QTLs (Supplementary Table S5).

A total of 12 QTLs for fibre quality were distributed on six chromosomes (Supplementary Fig. S1), and explained 7.59–37.09% of the PV, with LOD scores ranging from 4.04 to 9.57 (Supplementary Table S5). Of the 12 QTLs, 3 QTLs were for FL, 2 QTLs for FS, 2 QTLs for FE, 5 QTLs for MV, and 9 QTLs were novel (Supplementary Table S5).

### 3.9. Homology analysis between upland cotton and the A_2_ and D_5_ genomes

**Figure 8.**
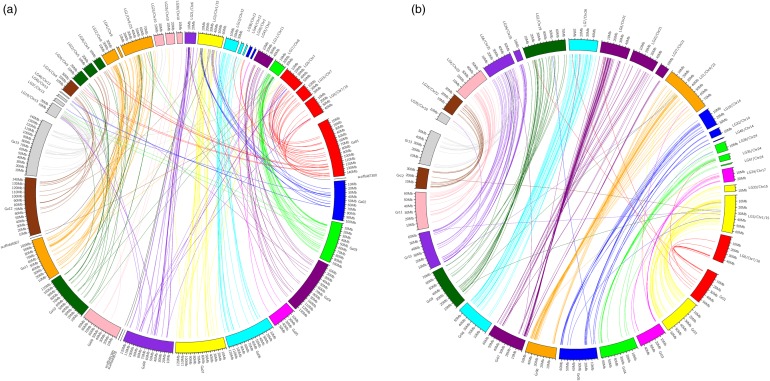
The distribution of 562 markers on the A_2_ and D_5_ genomes. (a) Analysis of homology between the A_T_ and A_2_ genomes; (b) analysis of homology between the D_T_ and D_5_ genomes.

The sequences of 562 markers (the sequences of SRAPs were not available) mapped on chromosomes of the upland cotton genetic map in this study were used to BLASTN with the diploid A_2_ genome of *G. arboretum* and the D_5_ genome of *G. raimondii*, using an *E*-value cut-off of 1e−10. After analysis, the homology and collinearity between the A_T_ genome and the A_2_ genome were high, except for Chr2 and Ga2, Chr5 and Ga10, and Chr10 and Ga9 (Fig. 8a; Supplementary Table S6a). In the current study, four unassembled scaffolds of the A_2_ genome were anchored by our map. HAU-DJ-S078 matched on scaffold7300, NAU2687 on scaffold3678, HAU-DJ-S168 on scaffold1365, and NBRI_HQ527767 on scaffold4507. Results showed that the homology and collinearity between the D_T_ genome and the D_5_ genome were high on every chromosome (Fig. 8b; Supplementary Table S6b).

## 4. Discussion

Until this study, there was no reference genome available for the allotetraploid cotton. The lack of genome sequences resulted in slow genome research progress in allotetraploid cotton, especially *G. hirsutum*. As the most important cultivated cotton, genome research of *G. hirsutum* is particularly urgent. In this study, the assembly produced 251,816 contigs, an amount that was too high, indicating that *Eco*RI restriction sites were abundant on the genome of *G. hirsutum*. There were more contigs for *G. hirsutum* than for other plants in an assembly analysis of PE RAD-seq studies.^17,38,42^ The sequencing depth, the assembly method, the high ploidy, and the heterozygosity of *G. hirsutum*, the large genome, and the high homology between the A_T_ genome and the D_T_ genome may all have contributed to larger numbers of assembled contigs.

There are many challenges for RAD-seq read assembly. Repetitive sequences are nearly indistinguishable in the context of a short sequence read. Short overlaps are easily pieced together in whole-genome assembly, even though short reads have a high error rate.^43^ When assembling the reads with Velvet,^19^ the user should choose parameters such as hash length and expected coverage. During assembly, a median value for each parameter is chosen, and the assembly therefore is less optimal in those regions that differ from that median. Velvet can also be reconfigured to use longer hash length than the default maximum of 31, but this requires hundreds of gigabytes of memory, which is a problem for whole-genome shotgun assembly of complex genomes such as allotetraploid cotton.

In this study, 10.33 Gb of clean reads of *G. hirsutum* were obtained from a pair of mapping parents using RAD-seq, and the GC contents of the two RAD libraries for DH962 and Jimian5 were 34.00 and 34.17%, respectively. The data sizes from the two materials were similar, and GC content was consistent with results from PE RAD-seq studies in other plant genomes.^17^ After identifying the sequences in common between the mapping parents using the CAP3 program^20^ (using the parameters of an overlap length cut-off of 80 bp and an overlap percent cut-off of 95), a Gh-D-J data set of ≈85.76 Mb was obtained. This indicated that the sequences from two genotypes represented ∼3.43% of the tetraploid cotton genome. So far, comparative sequencing of such a large part of the *G. hirsutum* genome has not been performed in two genotypes. These genome sequences will provide significant information for the molecular breeding project and genome research of *G. hirsutum*.

Fibre development has always been a major focus for cotton genome studies and breeders. In this study, 58 contigs were associated with the MYB family, 10 contigs with the expansin family, and 27 contigs with the ethylene response. The MYB family, expansin family, and ethylene biosynthesis are highly related to the initiation and elongation of cotton fibre cells.^44,45^ Another 17 contigs were found to be related to cellulose synthase (*CESA*) genes and 32 to cellulose synthase-like (*CSL*) genes. In the study by Yoo and Wendel,^46^ *CESA* and *CSL* genes were shown to be expressed specifically during primary and secondary cell wall biosynthesis of *G. hirsutum*. The genes found in the current study would be very useful for research of cotton fibre cell development. We also found 46 contigs that were related to NBS domains. In studies of the *G. arboretum* (A_2_) genome and the *G. raimondii* (D_5_) genome,^47,48^ NBS domains may be related with a Verticillium wilt resistance gene, so the discovery of NBS domains in the *G. hirsutum* genome is significant for research into Verticillium wilt resistance of *G. hirsutum*.

The two plant materials, DH962 and Jimian5, had quite different characteristics in yield and fibre quality traits,^3^ which were suitable for mapping population establishment. To develop genetic markers efficiently, the genomes of the two parents were sequenced. The number of hits was slightly less on Ga2 and Ga5 of the A_2_ genome (two shorter chromosomes)^48^ and on Gr3 and Gr12 of the D_5_ genome than on other chromosomes (two shorter chromosomes)^47^ (Fig. 6). The markers obtained here were uniformly distributed on every chromosome, and the quality of data from the RAD-seq was high and representative. The conditions were suitable for effective marker development.

Comparing the genome sequences of the two parents, a total of 17,138 SNPs were found in the two RAD libraries, and the transition/transversion ratio of all of the SNPs was 1.76. This result was consistent with other studies using RAD-seq.^17,38^ Byers *et al.*^11^ found 11,834 SNPs between two *G. hirsutum* varieties (Acala Maxxa and TX2094) from the GR-RSC libraries using the Roche 454 pyrosequencing platform. In the study by Srivastava *et al.*,^49^ a total of 1,440 expressed SSRs and 2,608 SNPs were identified from the transcriptome of two genotypes of *G. hirsutum* using the Roche 454 pyrosequencing platform. Because of the low levels of genetic diversity in upland cotton, there were very few polymorphisms between intraspecific strains of upland cotton. Analysis of upland cotton data involved some challenges: (i) upland cotton is an allotetraploid, which leads to the existence of lots of homologous sequences, (ii) a big complex genome structure (≈2.5 Gb), with too many repeat sequences, and (iii) lack of a reference genome. Here, 9,366 SNPs and 3,838 InDels suitable for further analysis were observed. Another 1,323 SSRs were also identified. All of the 14,527 markers were used for genome research, construction of a genetic map, and to explain the origin and evolution of cotton further. In this research, the number of detected SNPs was more than that found by Byers *et al.*^11^ and Srivastava *et al.*^49^ Until now, research related to SNP identification and genome structure in cotton has been far more superficial than in other major crops. Next-generation RAD sequencing was used here to detect SNPs in *G. hirsutum*. Results demonstrated that RAD-seq is an effective and economic method for SNP detection in *G. hirsutum*.

After validation by the parents, DH962 and Jimian5, the polymorphism rate of SSRs was found to be 6.27%. In previous studies,^3,6,50^ polymorphism rates of SSRs were ∼2.5–3.5%, and the rate of polymorphism markers from previous studies^28–34^ was 3.55% in the current study. In this study, markers were developed based on parental RAD-seq methodology, and the rate of polymorphism of SSRs was double that of previous studies. The rate of polymorphism of InDels and SNPs developed using the same method was 25.62 and 11.11%, respectively. These results indicated that this method of analysis could effectively and cost-effectively improve the detection of polymorphisms, and that these polymorphisms could be used as references for later marker development. In this study, 2× sequencing depth and 2 genotypes were used to develop markers. As the amount of sequencing material and depth increased, more data and more markers became available. At the same time, the limitations of bioinformatic tools and of genotyping technology can influence these results. To confirm universality and compatibility, all of the primer pairs were also screened between *G. hirsutum* cv. Emian22 and *G. barbadense* acc. 3-79, and revealed additional polymorphisms. The current approach was found to be effective with SSRs, InDels, and SNPs, and the polymorphic markers could be used to enrich the genetic map for further analysis.^41^ The calculated polymorphism rate in this study indicated that the application of InDels and SNPs could be more effective than SSRs in intraspecific population studies of upland cotton, and the use of markers found to be more effective in interspecific populations than in intraspecific populations. Owing to the narrow genetic base in upland cotton, the differences among upland cotton genome sequences may be mainly single-base deletions, insertions, and mutations. This phenomenon led to high polymorphism, so larger scale development of InDels and SNPs will be very informative in studying the molecular and quantitative genetics of cotton.

In this study, 535 polymorphic loci were identified, and a genetic map of upland cotton containing 1,013 loci was constructed. This map was one of two maps composed of >1,000 markers in upland cotton.^5^ The more saturated map provided a better platform to provide insight into and to analyse the complex cotton genome. We used the new map for mapping QTLs for yield components, 21 QTLs were detected and mapped on seven chromosomes, and 12 QTLs related to fibre quality were distributed on six chromosomes. In a previous work by Lin *et al.*,^18^ nine QTLs were detected for yield traits and five for fibre quality traits. Six QTLs were found on the adjacent area of the previous study, and another eight QTLs were detected, but the LOD value was decreased. Another 27 new QTLs were detected. The QTL *qFL-c10* was detected in the Lin *et al.*^18^ study and in the present study, and had a high LOD value, with >35% of the PV explained. This QTL may be a major loci controlling fibre length. Eight QTLs for LW and SI were found from 47.7 to 56.88 cM on LG17/Chr8 and explained 9.80–14.09% of the PV (Supplementary Table S5). As this region may be a hotspot for LW and SI, we will structure a permanent intraspecific population to verify the stability of these QTLs. As map density increased, the efficiency of QTL detection also increased considerably. The more saturated map can facilitate whole-genome sequencing, fine mapping of QTLs, and map-based cloning, and therefore a better understanding of cotton genome structure and improvement of cotton breeding.

The current results revealed that the homology and collinearity between A_T_ and A_2_ and D_T_ and D_5_ were high on every chromosome except for Chr2 and Ga2, Chr5 and Ga10, and Chr10 and Ga9. In previous reports,^41,51^ there was considerable homology and collinearity between the D_T_ of the allotetraploid cotton and the D_5_ genome. The current study is consistent with this result. In the A_T_ genome, some exchanges occurred between Ga10 and Ga12 and this phenomenon as observed in previous studies.^41,51^ At the same time, some fragments of Ga13 had introgressed into Gh10, and some fragments of Ga1 and Ga5 had introgressed into Gh2. This information provides an important reference for the sequence assembly of upland cotton and helps to better understand the origin and evolution of polyploidization and genomic integration studies in cotton. Because the genome data size of A_2_ is double that of D_5_, more errors would occur during assembly. High-density genetic linkage maps are often used to anchor scaffolds or contigs generated by whole-genome sequencing to chromosomes.^47,48^ Chen *et al.*^52^ assigned 44 unassembled scaffolds to diploid *B. rapa* chromosomes using the high-density genetic map of the allotetraploid *B. napus*. In this study, four unassembled scaffolds were anchored by the map produced here. The high homology and collinearity on these corresponding chromosomes between the A_T_ and A_2_ genomes indicated that the current results were appropriate for the refinement of A_2_ genome sequences. To prevent problems in the future, more markers are needed to enrich the genetic map to improve research into cotton genome structure.

## 5. Availability

The genome sequences of DH962 and Jimian5 developed from RAD-seq were available at NCBI Sequence Read Archive under SRA Project number SRP050345.

## Supplementary Data

Supplementary Data are available at www.dnaresearch.oxfordjournals.org.

## Funding

This work was financially supported by the National Basic Research Program (Grant No. 2011CB109303) and the Fundamental Research Funds for the Central Universities (Grant No. 2014PY015). Funding to pay the Open Access publication charges for this article was provided by the National Basic Research Program.

## Supplementary Material

Supplementary Data
