# Supplementary material for: Enrichment of an intraspecific genetic map of upland cotton by developing markers using parental RAD sequencing
Source: DNA Res. 2015 Feb 5;22(2):147–60. doi: 10.1093/dnares/dsu047 (PMC4401325; doi:10.1093/dnares/dsu047)
Supplement: Supplementary Data [file supp_22_2_147__index.html]

Enrichment of an intraspecific genetic map of upland cotton by developing markers using parental RAD sequencing — Enrichment of an intraspecific genetic map of upland cotton by developing markers using parental RAD sequencing — Enrichment of an intraspecific genetic map of upland cotton by developing markers using parental RAD sequencing — Supplementary Data 

# Enrichment of an intraspecific genetic map of upland cotton by developing markers using parental RAD sequencing

## Supplementary Data

Supplementary Data

**Files in this Data Supplement:**

- Supplementary Figure 1 - pdf file
- Supplementary Table 5 - pdf file
- Supplementary Table 1 - xls file
- Supplementary Table 2 - xls file
- Supplementary Table 3 - xls file
- Supplementary Table 4 - xls file
- Supplementary Table 6 - xls file
