# Supplementary material for: Enrichment of an intraspecific genetic map of upland cotton by developing markers using parental RAD sequencing
Source: DNA Res. 2015 Feb 5;22(2):147–60. doi: 10.1093/dnares/dsu047 (PMC4401325; doi:10.1093/dnares/dsu047)
Supplement: Supplementary Data [file supp_dsu047_dsu047supp_fig1.pdf]

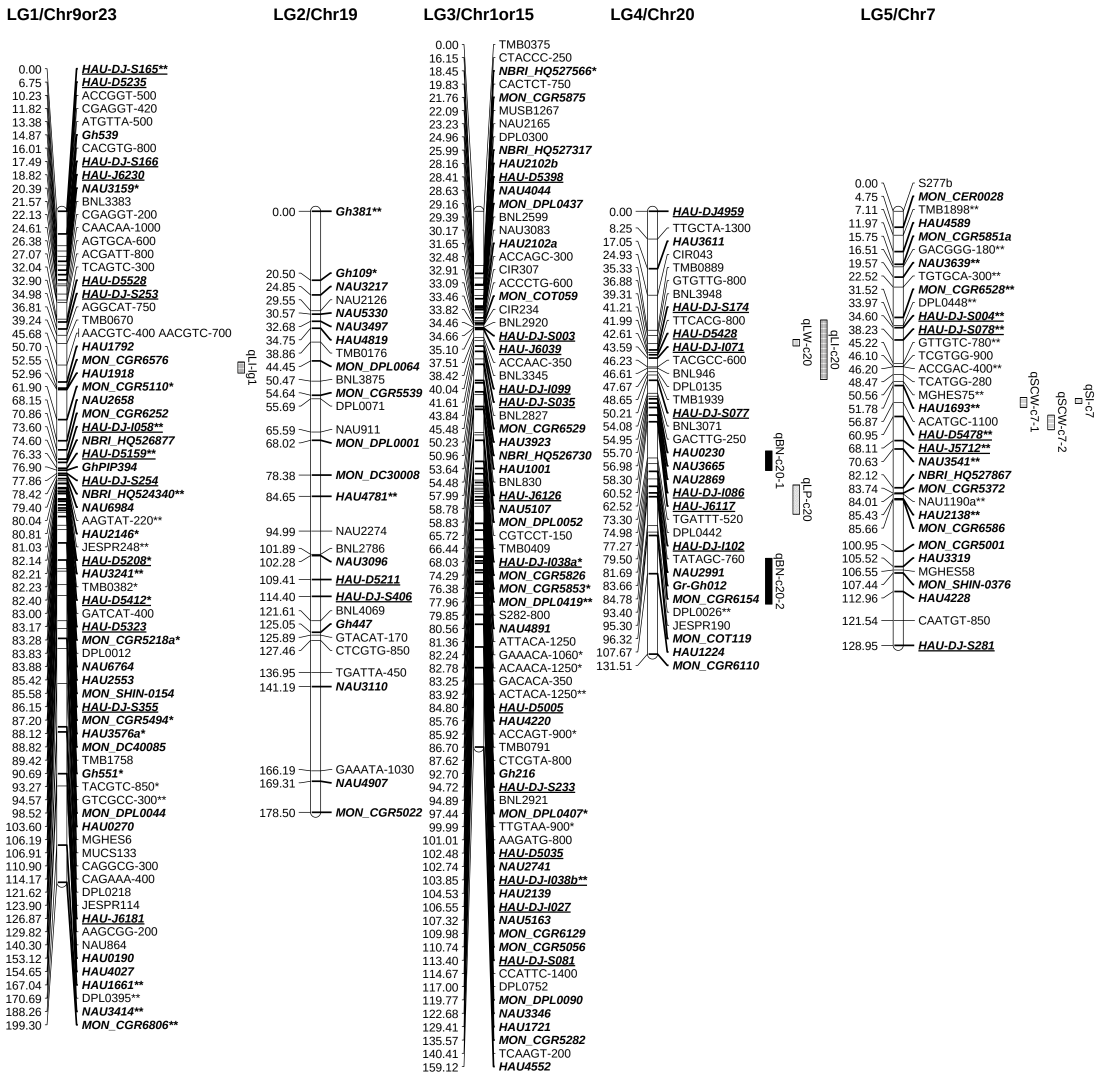

LG1/Chr9or23
0.00 HAU-DJ-S165**
6.75 HAU-D5235
10.23 ACCGGT-500
11.82 CGAGGT-420
13.38 ATGTTA-500
14.87 Gh539
16.01 CACGTG-800
17.49 HAU-DJ-S166
18.82 HAU-J6230
20.39 NAU3159*
21.57 BNL3383
22.13 CGAGGT-200
24.61 CAACAA-1000
26.38 AGTGCA-600
27.07 ACGATT-800
32.04 TCAGTC-300
32.90 HAU-D5528
34.98 HAU-DJ-S253
36.81 AGGCAT-750
39.24 TMB0670
45.68 AACGTC-400 AACGTC-700
50.70 HAU1792
52.55 MON_CGR6576
52.96 HAU1918
61.90 MON_CGR5110*
68.15 NAU2658
70.86 MON_CGR6252
73.60 HAU-DJ-I058**
74.60 NBRI_HQ526877
76.33 HAU-D5159**
76.90 GhPIP394
77.86 HAU-DJ-S254
78.42 NBRI_HQ524340**
79.40 NAU6984
80.04 AAGTAT-220**
80.81 HAU2146*
81.03 JESPR248**
82.14 HAU-D5208*
82.21 HAU3241**
82.23 TMB0382*
82.40 HAU-D5412*
83.00 GATCAT-400
83.17 HAU-D5323
83.28 MON_CGR5218a*
83.83 DPL0012
83.88 NAU6764
85.42 HAU2553
85.58 MON_SHIN-0154
86.15 HAU-DJ-S355
87.20 MON_CGR5494*
88.12 HAU3576a*
88.82 MON_DC40085
89.42 TMB1758
90.69 Gh551*
93.27 TACGTC-850*
94.57 GTCGCC-300**
98.52 MON_DPL0044
103.60 HAU0270
106.19 MGHES6
106.91 MUCS133
110.90 CAGGCG-300
114.17 CAGAAA-400
121.62 DPL0218
123.90 JESPR114
126.87 HAU-J6181
129.82 AAGCGG-200
140.30 NAU864
153.12 HAU0190
154.65 HAU4027
167.04 HAU1661**
170.69 DPL0395**
188.26 NAU3414**
199.30 MON_CGR6806**
qLI-lg1
LG2/Chr19
0.00 Gh381**
20.50 Gh109*
24.85 NAU3217
29.55 NAU2126
30.57 NAU5330
32.68 NAU3497
34.75 HAU4819
38.86 TMB0176
44.45 MON_DPL0064
50.47 BNL3875
54.64 MON_CGR5539
55.69 DPL0071
65.59 NAU911
68.02 MON_DPL0001
78.38 MON_DC30008
84.65 HAU4781**
94.99 NAU2274
101.89 BNL2786
102.28 NAU3096
109.41 HAU-D5211
114.40 HAU-DJ-S406
121.61 BNL4069
125.05 Gh447
125.89 GTACAT-170
127.46 CTCGTG-850
136.95 TGATTA-450
141.19 NAU3110
166.19 GAAATA-1030
169.31 NAU4907
178.50 MON_CGR5022
LG3/Chr1or15
0.00 TMB0375
16.15 CTACCC-250
18.45 NBRI_HQ527566*
19.83 CACTCT-750
21.76 MON_CGR5875
22.09 MUSB1267
23.23 NAU2165
24.96 DPL0300
25.99 NBRI_HQ527317
28.16 HAU2102b
28.41 HAU-D5398
28.63 NAU4044
29.16 MON_DPL0437
29.39 BNL2599
30.17 NAU3083
31.65 HAU2102a
32.48 ACCAGC-300
32.91 CIR307
33.09 ACCCTG-600
33.46 MON_COT059
33.82 CIR234
34.46 BNL2920
34.66 HAU-DJ-S003
35.10 HAU-J6039
37.51 ACCAAC-350
38.42 BNL3345
40.04 HAU-DJ-I099
41.61 HAU-DJ-S035
43.84 BNL2827
45.48 MON_CGR6529
50.23 HAU3923
50.96 NBRI_HQ526730
53.64 HAU1001
54.48 BNL830
57.99 HAU-J6126
58.78 NAU5107
58.83 MON_DPL0052
65.72 CGTCCT-150
66.44 TMB0409
68.03 HAU-DJ-I038a*
74.29 MON_CGR5826
76.38 MON_CGR5853*
77.96 MON_DPL0419**
79.85 S282-800
80.56 NAU4891
81.36 ATTACA-1250
82.24 GAAACA-1060*
82.78 ACAACA-1250*
83.25 GACACA-350
83.92 ACTACA-1250**
84.80 HAU-D5005
85.76 HAU4220
85.92 ACCAGT-900*
86.70 TMB0791
87.62 CTCGTA-800
92.70 Gh216
94.72 HAU-DJ-S233
94.89 BNL2921
97.44 MON_DPL0407*
99.99 TTGTAA-900*
101.01 AAGATG-800
102.48 HAU-D5035
102.74 NAU2741
103.85 HAU-DJ-I038b**
104.53 HAU2139
106.55 HAU-DJ-I027
107.32 NAU5163
109.98 MON_CGR6129
110.74 MON_CGR5056
113.40 HAU-DJ-S081
114.67 CCATTC-1400
117.00 DPL0752
119.77 MON_DPL0090
122.68 NAU3346
129.41 HAU1721
135.57 MON_CGR5282
140.41 TCAAGT-200
159.12 HAU4552
LG4/Chr20
0.00 HAU-DJ4959
8.25 TTGCTA-1300
17.05 HAU3611
24.93 CIR043
35.33 TMB0889
36.88 GTGTTG-800
39.31 BNL3948
41.21 HAU-DJ-S174
41.99 TTCACG-800
42.61 HAU-D5428
43.59 HAU-DJ-I071
46.23 TACGCC-600
46.61 BNL946
47.67 DPL0135
48.65 TMB1939
50.21 HAU-DJ-S077
54.08 BNL3071
54.95 GACTTG-250
55.70 HAU0230
56.98 NAU3665
58.30 NAU2869
60.52 HAU-DJ-I086
62.52 HAU-J6117
73.30 TGATTT-520
74.98 DPL0442
77.27 HAU-DJ-I102
79.50 TATAGC-760
81.69 NAU2991
83.66 Gr-Gh012
84.78 MON_CGR6154
93.40 DPL0026**
95.30 JESPR190
96.32 MON_COT119
107.67 HAU1224
131.51 MON_CGR6110
qBN-c20-1
qBN-c20-2
qLP-c20
qLW-c20
qLI-c20
LG5/Chr7
0.00 S277b
4.75 MON_CER0028
7.11 TMB1898**
11.97 HAU4589
15.75 MON_CGR5851a
16.51 GACGGG-180**
19.57 NAU3639**
22.52 TGTGCA-300**
31.52 MON_CGR6528**
33.97 DPL0448**
34.60 HAU-DJ-S004**
38.23 HAU-DJ-S078**
45.22 GTTGTC-780**
46.10 TCGTGG-900
46.20 ACCGAC-400**
48.47 TCATGG-280
50.56 MGHES75**
51.78 HAU1693**
56.87 ACATGC-1100
60.95 HAU-D5478**
68.11 HAU-J5712**
70.63 NAU3541**
82.12 NBRI_HQ527867
83.74 MON_CGR5372
84.01 NAU1190a**
85.43 HAU2138**
85.66 MON_CGR6586
100.95 MON_CGR5001
105.52 HAU3319
106.55 MGHES58
107.44 MON_SHIN-0376
112.96 HAU4228
121.54 CAATGT-850
128.95 HAU-DJ-S281
qSCW-c7-1
qSCW-c7-2
qSI-c7

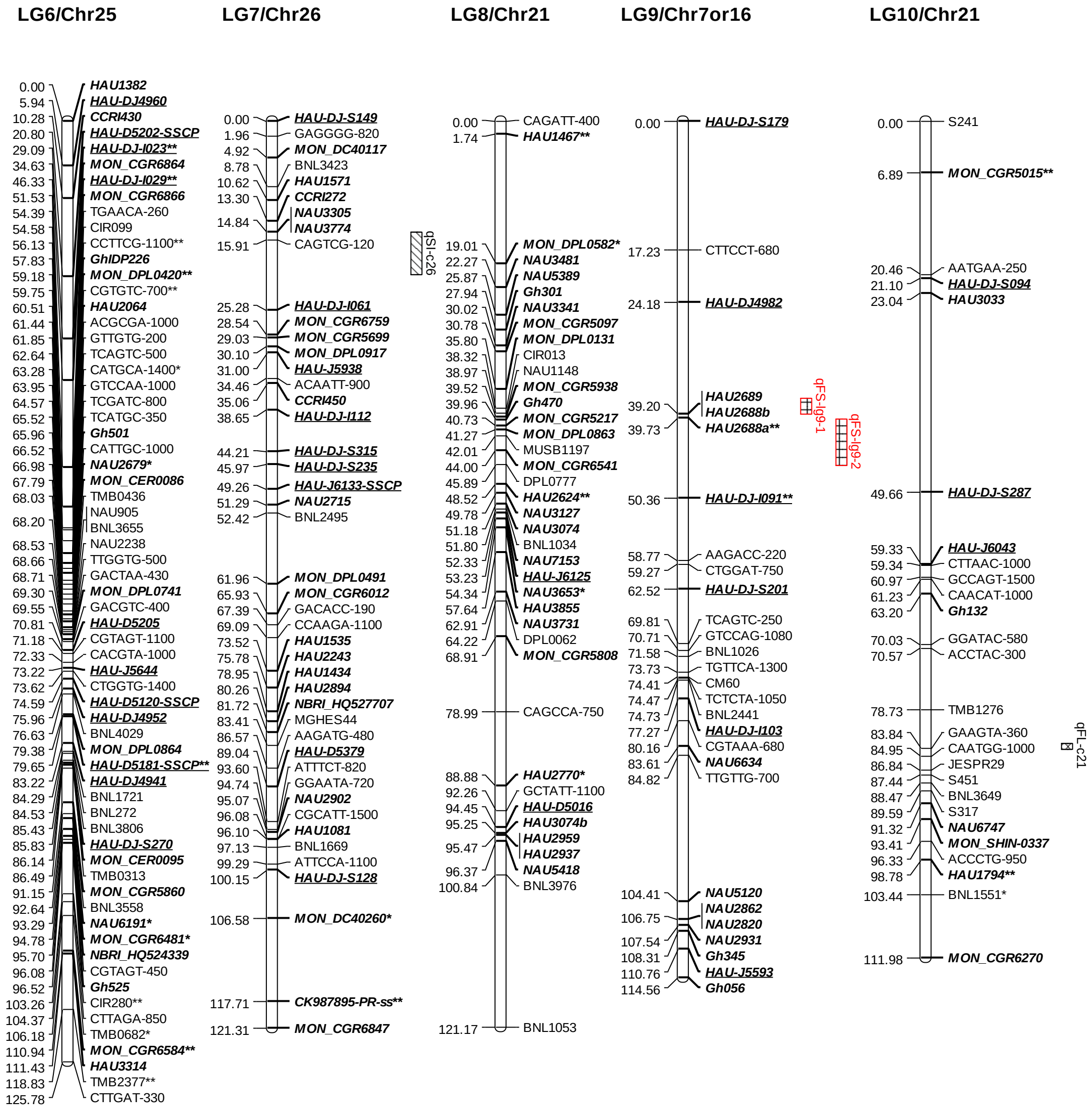
LG6/Chr25
0.00 HAU1382
5.94 HAU-DJ4960
10.28 CCRI430
20.80 HAU-D5202-SSCP
29.09 HAU-DJ-I023**
34.63 MON_CGR6864
46.33 HAU-DJ-I029**
51.53 MON_CGR6866
54.39 TGAACA-260
54.58 CIR099
56.13 CCTTCG-1100**
57.83 GhIDP226
59.18 MON_DPL0420**
59.75 CGTGTC-700**
60.51 HAU2064
61.44 ACGCGA-1000
61.85 GTTGTG-200
62.64 TCAGTC-500
63.28 CATGCA-1400*
63.95 GTCCAA-1000
64.57 TCGATC-800
65.52 TCATGC-350
65.96 Gh501
66.52 CATTGC-1000
66.98 NAU2679*
67.79 MON_CER0086
68.03 TMB0436
68.20 NAU905
BNL3655
68.53 NAU2238
68.66 TTGGTG-500
68.71 GACTAA-430
69.30 MON_DPL0741
69.55 GACGTC-400
70.81 HAU-D5205
71.18 CGTAGT-1100
72.33 CACGTA-1000
73.22 HAU-J5644
73.62 CTGGTG-1400
74.59 HAU-D5120-SSCP
75.96 HAU-DJ4952
76.63 BNL4029
79.38 MON_DPL0864
79.65 HAU-D5181-SSCP**
83.22 HAU-DJ4941
84.29 BNL1721
84.53 BNL272
85.43 BNL3806
85.83 HAU-DJ-S270
86.14 MON_CER0095
86.49 TMB0313
91.15 MON_CGR5860
92.64 BNL3558
93.29 NAU6191*
94.78 MON_CGR6481*
95.70 NBRI_HQ524339
96.08 CGTAGT-450
96.52 Gh525
103.26 CIR280**
104.37 CTTAGA-850
106.18 TMB0682*
110.94 MON_CGR6584**
111.43 HAU3314
118.83 TMB2377**
125.78 CTTGAT-330
LG7/Chr26
0.00 HAU-DJ-S149
1.96 GAGGGG-820
4.92 MON_DC40117
8.78 BNL3423
10.62 HAU1571
13.30 CCRI272
14.84 NAU3305
NAU3774
15.91 CAGTCG-120
qSI-c26
25.28 HAU-DJ-I061
28.54 MON_CGR6759
29.03 MON_CGR5699
30.10 MON_DPL0917
31.00 HAU-J5938
34.46 ACAATT-900
35.06 CCRI450
38.65 HAU-DJ-I112
44.21 HAU-DJ-S315
45.97 HAU-DJ-S235
49.26 HAU-J6133-SSCP
51.29 NAU2715
52.42 BNL2495
61.96 MON_DPL0491
65.93 MON_CGR6012
67.39 GACACC-190
69.09 CCAAGA-1100
73.52 HAU1535
75.78 HAU2243
78.95 HAU1434
80.26 HAU2894
81.72 NBRI_HQ527707
83.41 MGHES44
86.57 AAGATG-480
89.04 HAU-D5379
93.60 ATTTCT-820
94.74 GGAATA-720
95.07 NAU2902
96.08 CGCATT-1500
96.10 HAU1081
97.13 BNL1669
99.29 ATTCCA-1100
100.15 HAU-DJ-S128
106.58 MON_DC40260*
117.71 CK987895-PR-ss**
121.31 MON_CGR6847
LG8/Chr21
0.00 CAGATT-400
1.74 HAU1467**
19.01 MON_DPL0582*
22.27 NAU3481
25.87 NAU5389
27.94 Gh301
30.02 NAU3341
30.78 MON_CGR5097
35.80 MON_DPL0131
38.32 CIR013
38.97 NAU1148
39.52 MON_CGR5938
39.96 Gh470
40.73 MON_CGR5217
41.27 MON_DPL0863
42.01 MUSB1197
44.00 MON_CGR6541
45.89 DPL0777
48.52 HAU2624**
49.78 HAU3127
51.18 NAU3074
51.80 BNL1034
52.33 NAU7153
53.23 HAU-J6125
54.34 NAU3653*
57.64 HAU3855
62.91 NAU3731
64.22 DPL0062
68.91 MON_CGR5808
78.99 CAGCCA-750
88.88 HAU2770*
92.26 GCTATT-1100
94.45 HAU-D5016
95.25 HAU3074b
95.47 HAU2959
HAU2937
96.37 NAU5418
100.84 BNL3976
121.17 BNL1053
LG9/Chr7or16
0.00 HAU-DJ-S179
17.23 CTTCCT-680
24.18 HAU-DJ4982
39.20 HAU2689
HAU2688b
39.73 HAU2688a**
qFS-lg9-1
qFS-lg9-2
50.36 HAU-DJ-I091**
58.77 AAGACC-220
59.27 CTGGAT-750
62.52 HAU-DJ-S201
69.81 TCAGTC-250
70.71 GTCCAG-1080
71.58 BNL1026
73.73 TGTTCA-1300
74.41 CM60
74.47 TCTCTA-1050
74.73 BNL2441
77.27 HAU-DJ-I103
80.16 CGTAAA-680
83.61 NAU6634
84.82 TTGTTG-700
104.41 NAU5120
106.75 NAU2862
NAU2820
107.54 NAU2931
108.31 Gh345
110.76 HAU-J5593
114.56 Gh056
LG10/Chr21
0.00 S241
6.89 MON_CGR5015**
20.46 AATGAA-250
21.10 HAU-DJ-S094
23.04 HAU3033
49.66 HAU-DJ-S287
59.33 HAU-J6043
59.34 CTTAAC-1000
60.97 GCCAGT-1500
61.23 CAACAT-1000
63.20 Gh132
70.03 GGATAC-580
70.57 ACCTAC-300
78.73 TMB1276
83.84 GAAGTA-360
84.95 CAATGG-1000
86.84 JESPR29
87.44 S451
88.47 BNL3649
89.59 S317
91.32 NAU6747
93.41 MON_SHIN-0337
96.33 ACCCTG-950
98.78 HAU1794**
103.44 BNL1551*
111.98 MON_CGR6270
qFL-c21

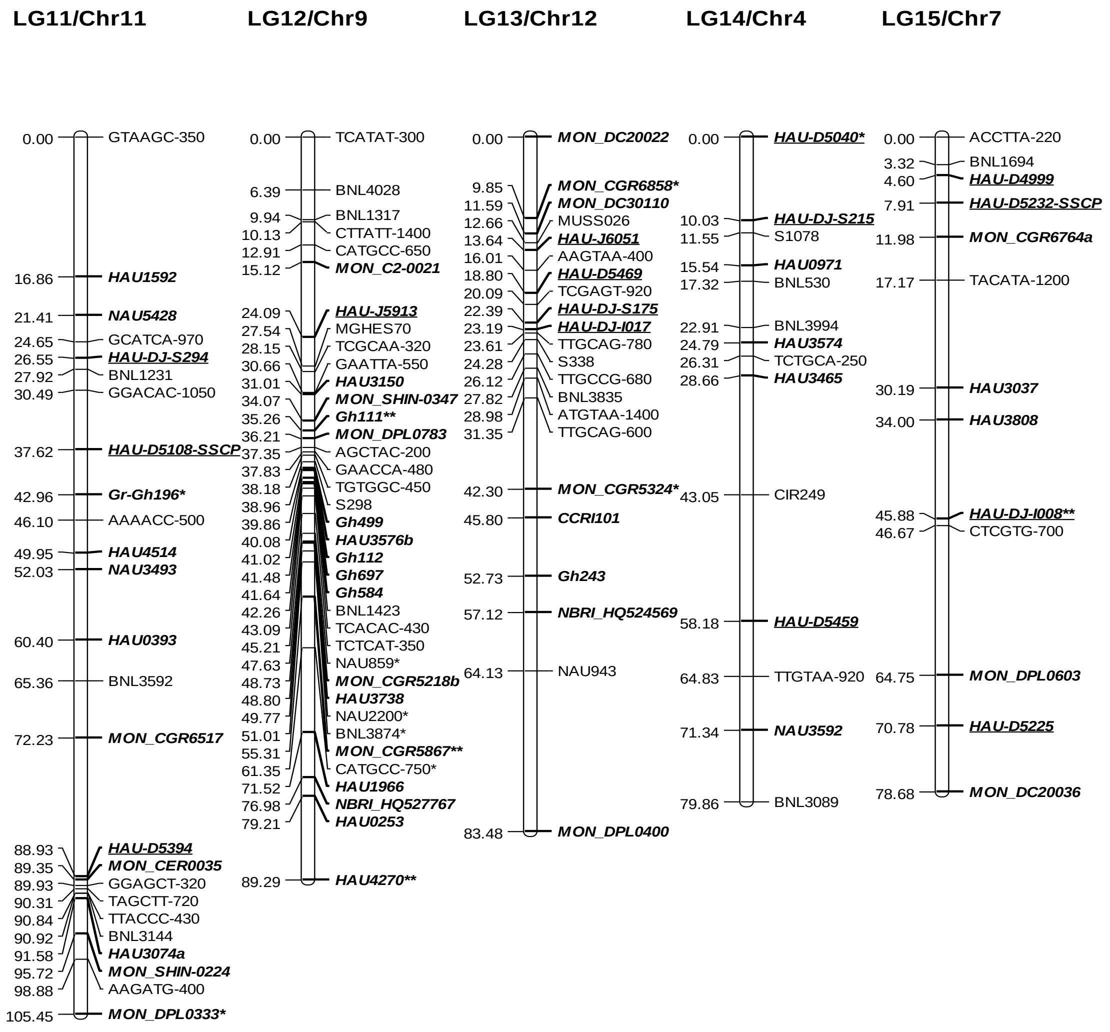

LG11/Chr11
LG12/Chr9
LG13/Chr12
LG14/Chr4
LG15/Chr7
0.00 GTAAGC-350
16.86 HAU1592
21.41 NAU5428
24.65 GCATCA-970
26.55 HAU-DJ-S294
27.92 BNL1231
30.49 GGACAC-1050
37.62 HAU-D5108-SSCP
42.96 Gr-Gh196*
46.10 AAAACC-500
49.95 HAU4514
52.03 NAU3493
60.40 HAU0393
65.36 BNL3592
72.23 MON_CGR6517
88.93 HAU-D5394
89.35 MON_CER0035
89.93 GGAGCT-320
90.31 TAGCTT-720
90.84 TTACCC-430
90.92 BNL3144
91.58 HAU3074a
95.72 MON_SHIN-0224
98.88 AAGATG-400
105.45 MON_DPL0333*
0.00 TCATAT-300
6.39 BNL4028
9.94 BNL1317
10.13 CTTATT-1400
12.91 CATGCC-650
15.12 MON_C2-0021
24.09 HAU-J5913
27.54 MGHES70
28.15 TCGCAA-320
30.66 GAATTA-550
31.01 HAU3150
34.07 MON_SHIN-0347
35.26 Gh111**
36.21 MON_DPL0783
37.35 AGCTAC-200
37.83 GAACCA-480
38.18 TGTGGC-450
38.96 S298
39.86 Gh499
40.08 HAU3576b
41.02 Gh112
41.48 Gh697
41.64 Gh584
42.26 BNL1423
43.09 TCACAC-430
45.21 TCTCAT-350
47.63 NAU859*
48.73 MON_CGR5218b
48.80 HAU3738
49.77 NAU2200*
51.01 BNL3874*
55.31 MON_CGR5867**
61.35 CATGCC-750*
71.52 HAU1966
76.98 NBRI_HQ527767
79.21 HAU0253
89.29 HAU4270**
0.00 MON_DC20022
9.85 MON_CGR6858*
11.59 MON_DC30110
12.66 MUSS026
13.64 HAU-J6051
16.01 AAGTAA-400
18.80 HAU-D5469
20.09 TCGAGT-920
22.39 HAU-DJ-S175
23.19 HAU-DJ-I017
23.61 TTGCAG-780
24.28 S338
26.12 TTGCCG-680
27.82 BNL3835
28.98 ATGTAA-1400
31.35 TTGCAG-600
42.30 MON_CGR5324*
45.80 CCRI101
52.73 Gh243
57.12 NBRI_HQ524569
64.13 NAU943
83.48 MON_DPL0400
0.00 HAU-D5040*
10.03 HAU-DJ-S215
11.55 S1078
15.54 HAU0971
17.32 BNL530
22.91 BNL3994
24.79 HAU3574
26.31 TCTGCA-250
28.66 HAU3465
43.05 CIR249
58.18 HAU-D5459
64.83 TTGTAA-920
71.34 NAU3592
79.86 BNL3089
0.00 ACCTTA-220
3.32 BNL1694
4.60 HAU-D4999
7.91 HAU-D5232-SSCP
11.98 MON_CGR6764a
17.17 TACATA-1200
30.19 HAU3037
34.00 HAU3808
45.88 HAU-DJ-I008**
46.67 CTCGTG-700
64.75 MON_DPL0603
70.78 HAU-D5225
78.68 MON_DC20036

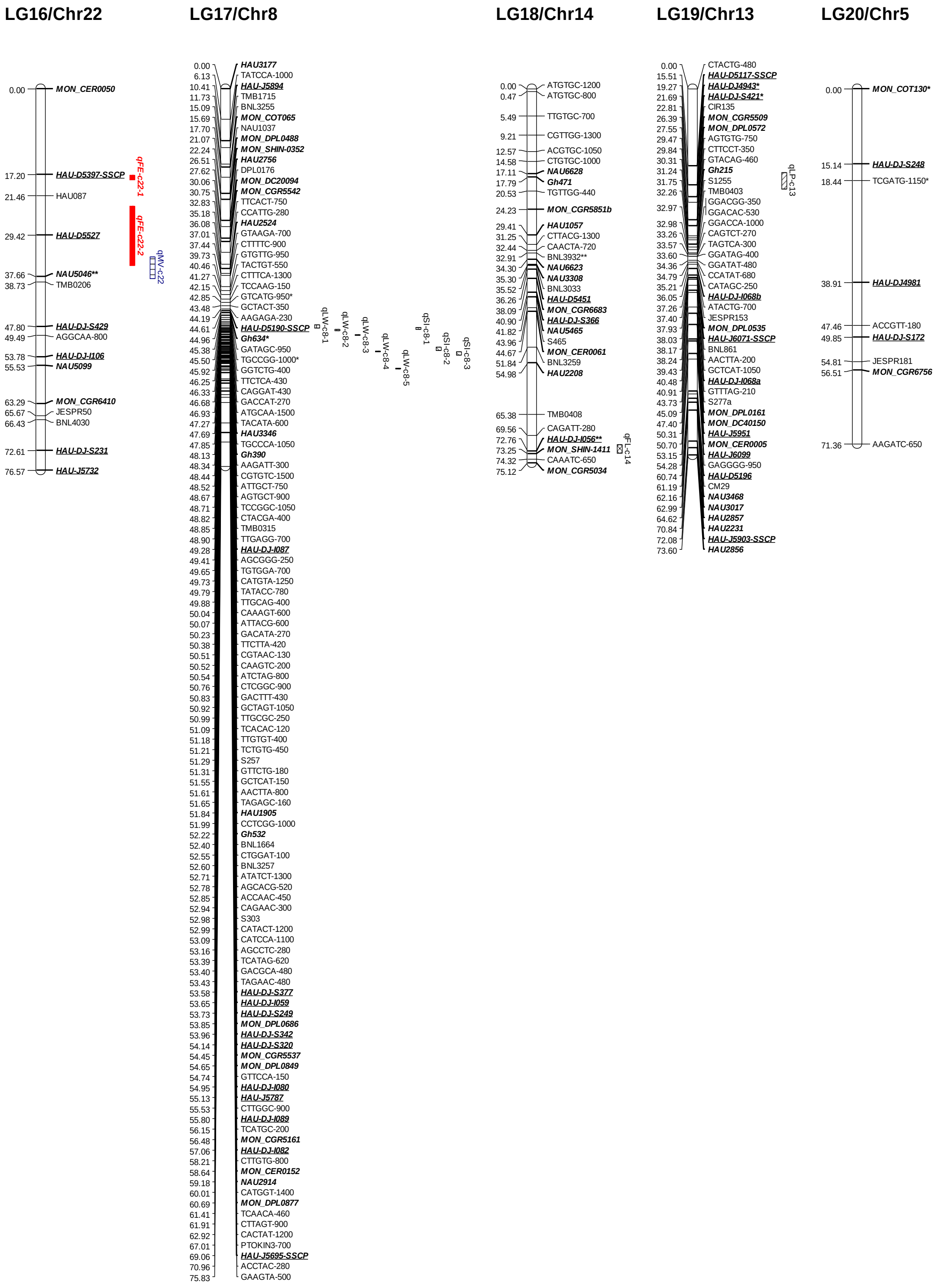

LG16/Chr22
0.00 MON_CER0050
17.20 HAU-D5397-SSCP
21.46 HAU087
29.42 HAU-D5527
37.66 NAU5046**
38.73 TMB0206
47.80 HAU-DJ-S429
49.49 AGGCAA-800
53.78 HAU-DJ-I106
55.53 NAU5099
63.29 MON_CGR6410
65.67 JESPR50
66.43 BNL4030
72.61 HAU-DJ-S231
76.57 HAU-J5732
qFE-c22-1
qFE-c22-2
qMV-c22
LG17/Chr8
0.00 HAU3177
6.13 TATCCA-1000
10.41 HAU-J5894
11.73 TMB1715
15.09 BNL3255
15.69 MON_COT065
17.70 NAU1037
21.07 MON_DPL0488
22.24 MON_SHIN-0352
26.51 HAU2756
27.62 DPL0176
30.06 MON_DC20094
30.75 MON_CGR5542
32.83 TTCACT-750
35.18 CCATTG-280
36.08 HAU2524
37.01 GTAAGA-700
37.44 CTTTTC-900
39.73 GTGTTG-950
40.46 TACTGT-550
41.27 CTTTCA-1300
42.15 TCCAAG-150
42.85 GTCATG-950*
43.48 GCTACT-350
44.19 AAGAGA-230
44.61 HAU-D5190-SSCP
44.96 Gh634*
45.38 GATAGC-950
45.50 TGCCCG-1000*
45.92 GGTCTG-400
46.25 TTCTCA-430
46.33 CAGGAT-430
46.68 GACCAT-270
46.93 ATGCAA-1500
47.27 TACATA-600
47.69 HAU3346
47.85 TGCCCA-1050
48.13 Gh390
48.34 AAGATT-300
48.44 CGTGTC-1500
48.52 ATTGCT-750
48.67 AGTGCT-900
48.71 TCCGGC-1050
48.82 CTACGA-400
48.85 TMB0315
48.90 TTGAGG-700
49.28 HAU-DJ-I087
49.41 AGCGGG-250
49.65 TGTGGA-700
49.73 CATGTA-1250
49.79 TATACC-780
49.88 TTGCAG-400
50.04 CAAAGT-600
50.07 ATTACG-600
50.23 GACATA-270
50.38 TTCTTA-420
50.51 CGTAAC-130
50.52 CAAGTC-200
50.54 ATCTAG-800
50.76 CTCGGC-900
50.83 GACTTT-430
50.92 GCTAGT-1050
50.99 TTGCGC-250
51.09 TCACAC-120
51.18 TTGTGT-400
51.21 TCTGTG-450
51.29 S257
51.31 GTTCTG-180
51.55 GCTCAT-150
51.61 AACTTA-800
51.65 TAGAGC-160
51.84 HAU1905
51.99 CCTCGG-1000
52.22 Gh532
52.40 BNL1664
52.55 CTGGAT-100
52.60 BNL3257
52.71 ATATCT-1300
52.78 AGCACG-520
52.85 ACCAAC-450
52.94 CAGAAC-300
52.98 S303
52.99 CATACT-1200
53.09 CATCCA-1100
53.16 AGCCTC-280
53.39 TCATAG-620
53.40 GACGCA-480
53.43 TAGAAC-480
53.58 HAU-DJ-S377
53.65 HAU-DJ-I059
53.73 HAU-DJ-S249
53.85 MON_DPL0686
53.96 HAU-DJ-S342
54.14 HAU-DJ-S320
54.45 MON_CGR5537
54.65 MON_DPL0849
54.74 GTTCCA-150
54.95 HAU-DJ-I080
55.13 HAU-J5787
55.53 CTTGGC-900
55.80 HAU-DJ-I089
56.15 TCATGC-200
56.48 MON_CGR5161
57.06 HAU-DJ-I082
58.21 CTTGTG-800
58.64 MON_CER0152
59.18 NAU2914
60.01 CATGGT-1400
60.69 MON_DPL0877
61.41 TCAACA-460
61.91 CTTAGT-900
62.92 CACTAT-1200
67.01 PTOKIN3-700
69.06 HAU-J5695-SSCP
70.96 ACCTAC-280
75.83 GAAGTA-500
qLW-c8-1
qLW-c8-2
qLW-c8-3
qLW-c8-4
qLW-c8-5
qSI-c8-1
qSI-c8-2
qSI-c8-3
LG18/Chr14
0.00 ATGTGC-1200
0.47 ATGTGC-800
5.49 TTGTGC-700
9.21 CGTTGG-1300
12.57 ACGTGC-1050
14.58 CTGTGC-1000
17.11 NAU6628
17.79 Gh471
20.53 TGTTGG-440
24.23 MON_CGR5851b
29.41 HAU1057
31.25 CTTACG-1300
32.44 CAACTA-720
32.91 BNL3932**
34.30 NAU6623
35.30 NAU3308
35.52 BNL3033
36.26 HAU-D5451
38.09 MON_CGR6683
40.90 HAU-DJ-S366
41.82 NAU5465
43.96 S465
44.67 MON_CER0061
51.84 BNL3259
54.98 HAU2208
65.38 TMB0408
69.56 CAGATT-280
72.76 HAU-DJ-I056**
73.25 MON_SHIN-1411
74.32 CAAATC-650
75.12 MON_CGR5034
qFL-c14
LG19/Chr13
0.00 CTACTG-480
15.51 HAU-D5117-SSCP
19.27 HAU-DJ4943*
21.69 HAU-DJ-S421*
22.81 CIR135
26.39 MON_CGR5509
27.55 MON_DPL0572
29.47 AGTGTG-750
29.84 CTTCCT-350
30.31 GTACAG-460
31.24 Gh215
31.75 S1255
32.26 TMB0403
32.97 GGACGG-350
GGACAC-530
32.98 GGACCA-1000
33.26 CAGTCT-270
33.57 TAGTCA-300
33.60 GGATAG-400
34.36 GGATAT-480
34.79 CCATAT-680
35.21 CATAGC-250
36.05 HAU-DJ-I068b
37.26 ATACTG-700
37.40 JESPR153
37.93 MON_DPL0535
38.03 HAU-J6071-SSCP
38.17 BNL861
38.24 AACTTA-200
39.43 GCTCAT-1050
40.48 HAU-DJ-I068a
40.91 GTTTAG-210
43.73 S277a
45.09 MON_DPL0161
47.40 MON_DC40150
50.31 HAU-J5951
50.70 MON_CER0005
53.15 HAU-J6099
54.28 GAGGGG-950
60.74 HAU-D5196
61.19 CM29
62.16 NAU3468
62.99 NAU3017
64.62 HAU2857
70.84 HAU2231
72.08 HAU-J5903-SSCP
73.60 HAU2856
qLP-c13
LG20/Chr5
0.00 MON_COT130*
15.14 HAU-DJ-S248
18.44 TCGATG-1150*
38.91 HAU-DJ4981
47.46 ACCGTT-180
49.85 HAU-DJ-S172
54.81 JESPR181
56.51 MON_CGR6756
71.36 AAGATC-650

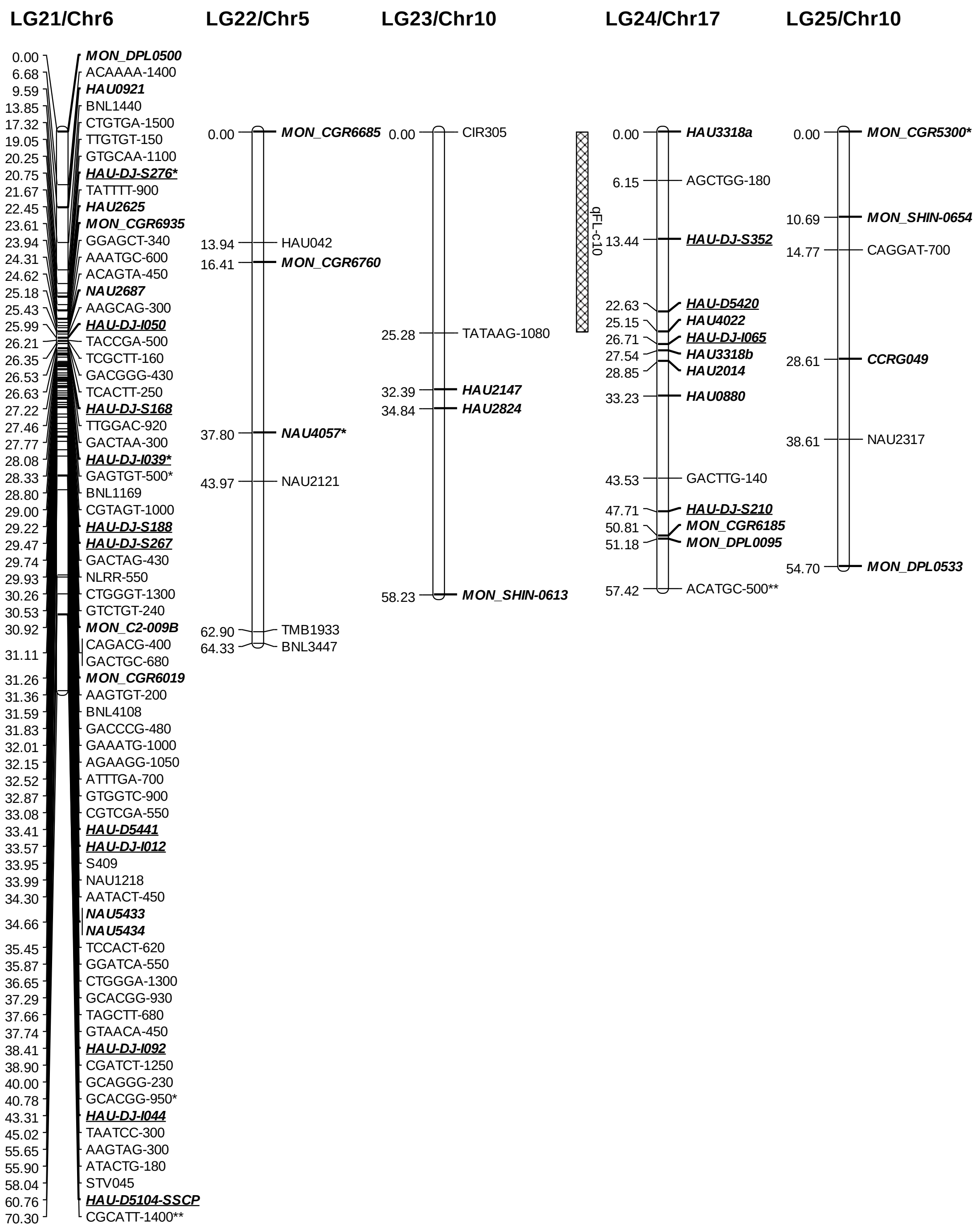
LG21/Chr6
0.00 MON_DPL0500
6.68 ACAAAA-1400
9.59 HAU0921
13.85 BNL1440
17.32 CTGTGA-1500
19.05 TTGTGT-150
20.25 GTGCAA-1100
20.75 HAU-DJ-S276*
21.67 TATTTT-900
22.45 HAU2625
23.61 MON_CGR6935
23.94 GGAGCT-340
24.31 AAATGC-600
24.62 ACAGTA-450
25.18 NAU2687
25.43 AAGCAG-300
25.99 HAU-DJ-I050
26.21 TACCGA-500
26.35 TCGCTT-160
26.53 GACGGG-430
26.63 TCACTT-250
27.22 HAU-DJ-S168
27.46 TTGGAC-920
27.77 GACTAA-300
28.08 HAU-DJ-I039*
28.33 GAGTGT-500*
28.80 BNL1169
29.00 CGTAGT-1000
29.22 HAU-DJ-S188
29.47 HAU-DJ-S267
29.74 GACTAG-430
29.93 NLRR-550
30.26 CTGGGT-1300
30.53 GTCTGT-240
30.92 MON_C2-009B
31.11 CAGACG-400
GACTGC-680
31.26 MON_CGR6019
31.36 AAGTGT-200
31.59 BNL4108
31.83 GACCCG-480
32.01 GAAATG-1000
32.15 AGAAGG-1050
32.52 ATTTGA-700
32.87 GTGGTC-900
33.08 CGTCGA-550
33.41 HAU-D5441
33.57 HAU-DJ-I012
33.95 S409
33.99 NAU1218
34.30 AATACT-450
34.66 NAU5433
NAU5434
35.45 TCCACT-620
35.87 GGATCA-550
36.65 CTGGGA-1300
37.29 GCACGG-930
37.66 TAGCTT-680
37.74 GTAACA-450
38.41 HAU-DJ-I092
38.90 CGATCT-1250
40.00 GCAGGG-230
40.78 GCACGG-950*
43.31 HAU-DJ-I044
45.02 TAATCC-300
55.65 AAGTAG-300
55.90 ATACTG-180
58.04 STV045
60.76 HAU-D5104-SSCP
70.30 CGCATT-1400**
LG22/Chr5
0.00 MON_CGR6685
13.94 HAU042
16.41 MON_CGR6760
37.80 NAU4057*
43.97 NAU2121
62.90 TMB1933
64.33 BNL3447
LG23/Chr10
0.00 CIR305
25.28 TATAAG-1080
32.39 HAU2147
34.84 HAU2824
58.23 MON_SHIN-0613
qFL-c10
LG24/Chr17
0.00 HAU3318a
6.15 AGCTGG-180
13.44 HAU-DJ-S352
22.63 HAU-D5420
25.15 HAU4022
26.71 HAU-DJ-I065
27.54 HAU3318b
28.85 HAU2014
33.23 HAU0880
43.53 GACTTG-140
47.71 HAU-DJ-S210
50.81 MON_CGR6185
51.18 MON_DPL0095
57.42 ACATGC-500**
LG25/Chr10
0.00 MON_CGR5300*
10.69 MON_SHIN-0654
14.77 CAGGAT-700
28.61 CCRG049
38.61 NAU2317
54.70 MON_DPL0533

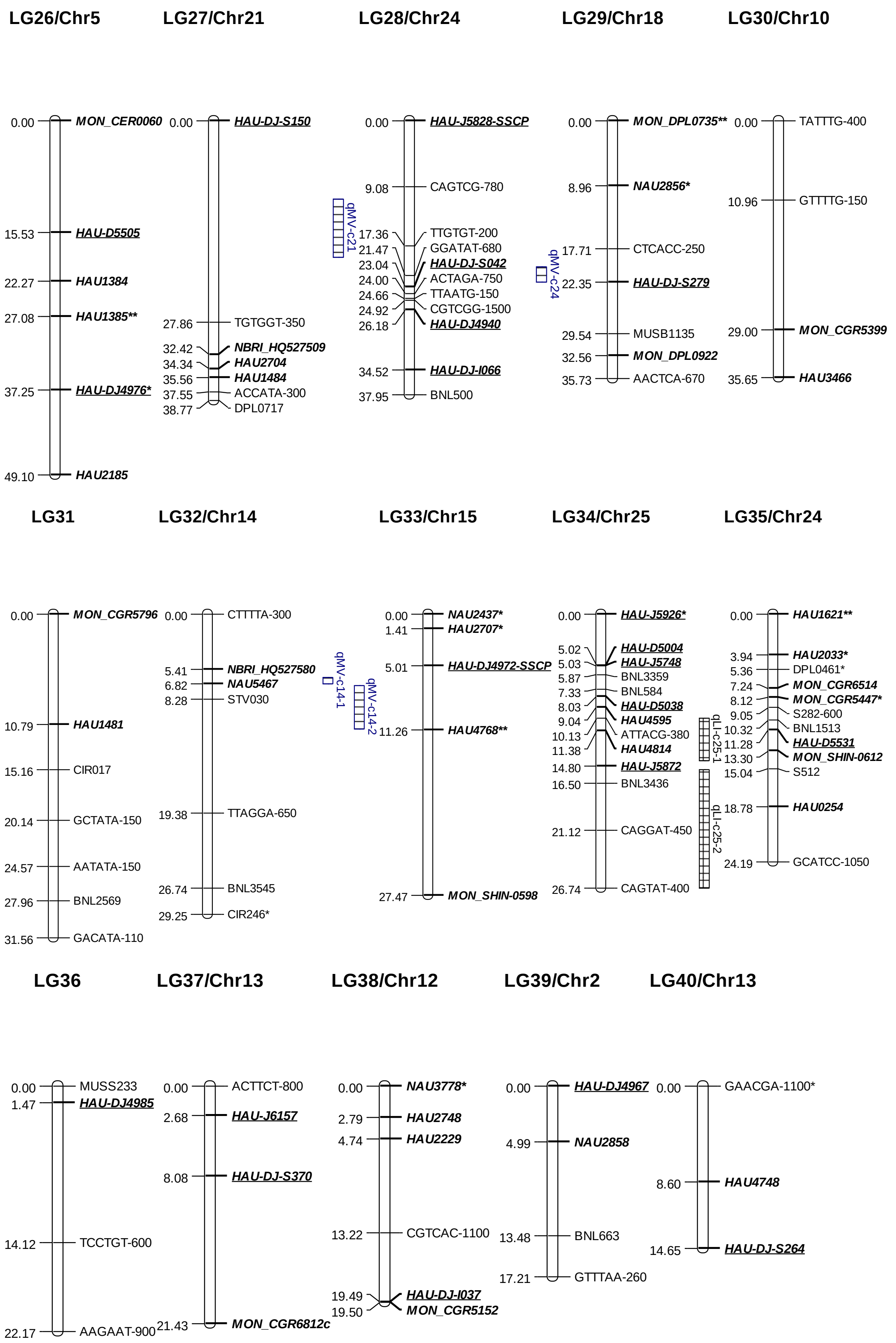
LG26/Chr5
LG27/Chr21
LG28/Chr24
LG29/Chr18
LG30/Chr10
0.00 MON_CER0060
15.53 HAU-D5505
22.27 HAU1384
27.08 HAU1385**
37.25 HAU-DJ4976*
49.10 HAU2185
0.00 HAU-DJ-S150
27.86 TGTGGT-350
32.42 NBRI_HQ527509
34.34 HAU2704
35.56 HAU1484
37.55 ACCATA-300
38.77 DPL0717
0.00 HAU-J5828-SSCP
9.08 CAGTCG-780
17.36 TTGTGT-200
21.47 GGATAT-680
23.04 HAU-DJ-S042
24.00 ACTAGA-750
24.66 TTAATG-150
24.92 CGTCGG-1500
26.18 HAU-DJ4940
34.52 HAU-DJ-I066
37.95 BNL500
qMV-c21
0.00 MON_DPL0735**
8.96 NAU2856*
17.71 CTCACC-250
22.35 HAU-DJ-S279
29.54 MUSB1135
32.56 MON_DPL0922
35.73 AACTCA-670
qMV-c24
0.00 TATTTG-400
10.96 GTTTTG-150
29.00 MON_CGR5399
35.65 HAU3466
LG31
LG32/Chr14
LG33/Chr15
LG34/Chr25
LG35/Chr24
0.00 MON_CGR5796
10.79 HAU1481
15.16 CIR017
20.14 GCTATA-150
24.57 AATATA-150
27.96 BNL2569
31.56 GACATA-110
0.00 CTTTTA-300
5.41 NBRI_HQ527580
6.82 NAU5467
8.28 STV030
19.38 TTAGGA-650
26.74 BNL3545
29.25 CIR246*
qMV-c14-1
qMV-c14-2
0.00 NAU2437*
1.41 HAU2707*
5.01 HAU-DJ4972-SSCP
11.26 HAU4768**
27.47 MON_SHIN-0598
0.00 HAU-J5926*
5.02 HAU-D5004
5.03 HAU-J5748
5.87 BNL3359
7.33 BNL584
8.03 HAU-D5038
9.04 HAU4595
10.13 ATTACG-380
11.38 HAU4814
14.80 HAU-J5872
16.50 BNL3436
21.12 CAGGAT-450
26.74 CAGTAT-400
qLI-c25-1
qLI-c25-2
0.00 HAU1621**
3.94 HAU2033*
5.36 DPL0461*
7.24 MON_CGR6514
8.12 MON_CGR5447*
9.05 S282-600
10.32 BNL1513
11.28 HAU-D5531
13.30 MON_SHIN-0612
15.04 S512
18.78 HAU0254
24.19 GCATCC-1050
LG36
LG37/Chr13
LG38/Chr12
LG39/Chr2
LG40/Chr13
0.00 MUSS233
1.47 HAU-DJ4985
14.12 TCCTGT-600
22.17 AAGAAT-900
0.00 ACTTCT-800
2.68 HAU-J6157
8.08 HAU-DJ-S370
21.43 MON_CGR6812c
0.00 NAU3778*
2.79 HAU2748
4.74 HAU2229
13.22 CGTCAC-1100
19.49 HAU-DJ-I037
19.50 MON_CGR5152
0.00 HAU-DJ4967
4.99 NAU2858
13.48 BNL663
17.21 GTTTAA-260
0.00 GAACGA-1100*
8.60 HAU4748
14.65 HAU-DJ-S264

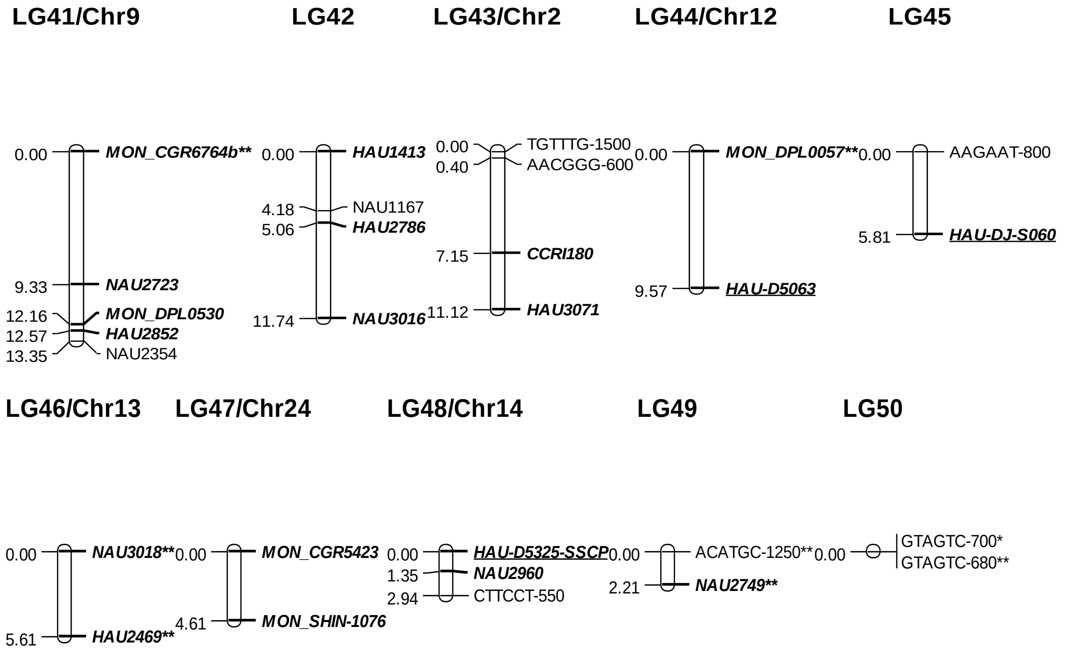

Figure S1. A genetic map of the cotton genome developed in 137 plants of the F_2 population derived from *G. hirsutum* accession DH962 and *G. hirsutum* cultivar Jimian5. A total of 1013 loci were mapped on 50 linkage groups. The locations of 21 QTLs for yield components, 12 QTLs for fiber quality are shown. Markers showing segregation distortion are indicated by asterisks (*P < 0.05; ** P < 0.01). The marker development from RAD-seq are underlined, italic, and in bold, other new added markers are italic, in bold.
