# Supplementary material for: Enrichment of an intraspecific genetic map of upland cotton by developing markers using parental RAD sequencing
Source: DNA Res. 2015 Feb 5;22(2):147–60. doi: 10.1093/dnares/dsu047 (PMC4401325; doi:10.1093/dnares/dsu047)
Supplement: Supplementary Data [file supp_dsu047_dsu047supp_table5.pdf]

QTLs detected for yield components and fiber quality in F_2 population of upland cotton. The new QTLs were in bold

Traits	QTL	Location	Marker interval	Nearest marker	Position	LOD	R^2	A	D	Mode
BN	**qBN-c20-1**	**Chr20**	**HAU-J6117-HAU-DJ-I102**	**DPL0442**	**74.31**	**5.17**	**14.55%**	**-1.57**	**0.08**	**A**
	**qBN-c20-2**	**Chr20**	**MON_COT119-MON_CGR6110**	**HAU1224**	**107.68**	**5.64**	**14.88%**	**1.92**	**-0.64**	**PD**
SCW	**qSCW-c7-1**	**Chr7**	**HAU1693-HAU-D5478**	**ACATGC-1100**	**56.79**	**4.49**	**11.61%**	**-0.09**	**0.22**	**OD**
	**qSCW-c7-2**	**Chr7**	**HAU-D5478-HAU-J5712**	**HAU-D5478**	**61.96**	**4.95**	**12.27%**	**0.06**	**0.42**	**OD**
LW	**qLW-c20**	**Chr20**	**GTGTTG-800-HAU-DJ-S174**	**BNL3948**	**38.89**	**4.67**	**11.50%**	**0.09**	**-0.01**	**A**
	qLW-c8-1	Chr8	TACATA-600-Gh390	HAU3346	47.7	4.78	11.04%	-0.06	0.02	PD
	**qLW-c8-2**	**Chr8**	**Gh390-AGTGCT-900**	**CGTGTC-1500**	**48.45**	**4.59**	**10.62%**	**-0.07**	**0.03**	**PD**
	**qLW-c8-3**	**Chr8**	**HAU-DJ-I087-TGTGGA-700**	**AGCGGG-250**	**49.42**	**4.44**	**10.41%**	**-0.06**	**0.02**	**PD**
	**qLW-c8-4**	**Chr8**	**BNL3257-AGCACG-520**	**ATATCT-1300**	**52.72**	**4.39**	**10.31%**	**-0.06**	**0.03**	**PD**
	**qLW-c8-5**	**Chr8**	**TCATGC-200-MON_CGR5161**	**TCATGC-200**	**56.16**	**4.22**	**9.80%**	**-0.06**	**0.03**	**PD**
SI	**qSI-c7**	**Chr7**	**HAU1693-HAU-D5478**	**ACATGC-1100**	**56.88**	**4.50**	**12.15%**	**-0.29**	**0.45**	**OD**
	**qSI-c26**	**Chr26**	**NAU3305-HAU-DJ-I061**	**CAGTCG-120**	**17.92**	**4.26**	**13.19%**	**0.37**	**-0.44**	**OD**
	**qSI-c8-1**	**Chr8**	**TGCCCA-1050-AAGATT-300**	**Gh390**	**48.14**	**5.01**	**14.09%**	**-0.31**	**-0.06**	**PD**
	qSI-c8-2	Chr8	TAGAGC-160-CTGGAT-100	CCTCGG-1000	52	4.96	13.91%	-0.36	0.00	A
	**qSI-c8-3**	**Chr8**	**AGCACG-520-TAGAAC-480**	**CATCCA-1100**	**53**	**4.28**	**12.26%**	**-0.31**	**-0.03**	**A**
LP	**qLP-c20**	**Chr20**	**TATAGC-760-DPL0026**	**Gr-Gh012**	**83.67**	**5.77**	**13.43%**	**0.92**	**-0.65**	**PD**
	**qLP-c13**	**Chr13**	**HAU-D5117-SSCP-HAU-DJ-S421**	**HAU-DJ4943**	**19.28**	**4.53**	**10.70%**	**-0.66**	**-0.35**	**PD**
LI	**qLI-lg1**	**LG1**	**TMB0670-HAU1792**	**AACGTC-400**	**45.69**	**4.49**	**10.16%**	**0.19**	**-0.10**	**PD**
	qLI-c20	Chr20	CIR043-HAU-DJ-S077	BNL3948	38.89	7.83	16.62%	0.24	-0.07	PD
	**qLI-c25-1**	**Chr25**	**HAU4595-HAU-J5872**	**HAU4814**	**11.39**	**6.14**	**12.41%**	**-0.23**	**0.03**	**PD**
	**qLI-c25-2**	**Chr25**	**HAU-J5872-CAGTAT-400**	**CAGGAT-450**	**21.13**	**6.91**	**14.35%**	**-0.25**	**0.11**	**PD**
FL	**qFL-c21**	**Chr21**	**TMB1276-CAATGG-1000**	**GAAGTA-360**	**83.74**	**4.11**	**7.59%**	**0.06**	**0.46**	**OD**
	**qFL-c14**	**Chr14**	**CAGATT-280-MON_SHIN-1411**	**HAU-DJ-I056**	**72.77**	**6.32**	**13.38%**	**0.62**	**-0.45**	**PD**
	qFL-c10	Chr10	CIR305-TATAAG-1080	CIR305	6.01	9.57	37.09%	1.16	1.15	D
FS	**qFS-lg9-1**	**LG9**	**HAU-DJ4982-HAU2688a**	**HAU2689**	**38.19**	**4.13**	**18.88%**	**0.51**	**1.06**	**OD**
	**qFS-lg9-2**	**LG9**	**HAU2688a-HAU-DJ-I091**	**HAU2688a**	**41.74**	**4.74**	**23.23%**	**0.57**	**1.76**	**OD**
FE	**qFE-c22-1**	**Chr22**	**HAU-D5397-SSCP-HAU087**	**HAU-D5397-SSCP**	**17.21**	**4.05**	**10.08%**	**-0.14**	**0.02**	**A**
	**qFE-c22-2**	**Chr22**	**HAU087-NAU5046**	**HAU-D5527**	**32.43**	**4.63**	**13.92%**	**-0.11**	**-0.13**	**D**
MV	**qMV-c22**	**Chr22**	**HAU-D5527-TMB0206**	**NAU5046**	**37.67**	**5.38**	**12.24%**	**0.06**	**0.18**	**OD**
	**qMV-c21**	**Chr21**	**HAU-DJ-S150-TGTGGT-350**	**TGTGGT-350**	**15.01**	**4.31**	**18.92%**	**0.01**	**0.23**	**OD**
	qMV-c24	Chr24	TTGTGT-200-HAU-DJ-S042	GGATAT-680	21.48	4.24	10.23%	-0.13	-0.04	PD
	qMV-c14-1	Chr14	NBRI_HQ527580-NAU5467	NAU5467	6.42	4.04	8.72%	0.00	-0.15	OD
	**qMV-c14-2**	**Chr14**	**NAU5467-TTAGGA-650**	**STV030**	**7.83**	**4.12**	**8.85%**	**-0.01**	**-0.15**	**OD**
